# The lariat RNA debranching enzyme DBR1 enhances HIF-1 signaling and chemoresistance at the expense of lariat RNA processing

**DOI:** 10.1016/j.jbc.2026.113484

**Published:** 2026-08-25

**Authors:** Phuong Thi Lien Nguyen, Minoru Kobayashi, Sho Koyasu, Kumi Johchi, Hiroshi Harada

**Affiliations:** 1 Laboratory of Cancer Cell Biology, Graduate School of Biostudies, Kyoto University, Kyoto, Japan; 2 Department of Genome Dynamics, Radiation Biology Center, Graduate School of Biostudies, Kyoto University, Kyoto, Japan; 3 Department of Diagnostic Imaging and Nuclear Medicine, Graduate School of Medicine, Kyoto University, Kyoto, Japan

**Keywords:** hypoxia, hypoxia-inducible factor 1, debranching RNA lariats 1, chemoresistance, RNA lariat debranching

## Abstract

Severely hypoxic microenvironments that induce cell-cycle arrest and chemoresistance are frequently found within solid tumors. Since hypoxia-inducible factor 1 (HIF-1) serves as a central transcription factor for cellular adaptation to oxygen deprivation, identification of novel regulators of HIF-1 activity may provide novel therapeutic opportunities. Here, we performed a genome-wide screening for novel positive regulators of HIF-1 and identified debranching RNA lariats 1 (DBR1). DBR1 was found to form a complex with the regulatory subunit of HIF-1, HIF-1α, promote its nuclear accumulation and recruitment to hypoxia-response elements, and enhance HIF-1-dependent transcription without altering *HIF1A* mRNA or protein levels. Notably, a catalytic mutant of DBR1 lacking debranching activity retained the ability to activate HIF-1. Interestingly, under hypoxic conditions, HIF-1 redirected DBR1 from its canonical RNA debranching function toward HIF-1-dependent transcriptional regulation, resulting in reduced debranching activity, lariat RNA accumulation, and impaired cell growth. Furthermore, DBR1 contributed to hypoxia-induced resistance to multiple conventional chemotherapeutic agents. Collectively, our findings support a model in which HIF-1 functionally redistributes DBR1 from lariat RNA processing toward HIF-1-dependent transcription, thereby promoting HIF-1 signaling and chemoresistance at the expense of lariat RNA processing.

A major challenge limiting chemotherapy efficiency is the presence of slow-dividing cancer cell populations within solid tumors (1, 2, 3, 4). These populations are largely shaped by hypoxia (5), a defining feature of the tumor microenvironment that emerges as rapidly growing tumors frequently outstrip developing vasculature (6). In response to hypoxic stress, cancer cells rewire transcriptional (7) and metabolic programs (8) to reduce biosynthetic activity and proliferation, thereby lowering metabolic demand. Although this adaptation promotes survival under oxygen limitation, it also reduces sensitivity to chemotherapeutic agents, which typically target proliferating cancer cells, thereby contributing to tumor progression and therapy resistance (1, 2, 3, 4).

Many of the adaptive responses that enhance viability and promote chemotherapy resistance in hypoxic tumors are coordinated by hypoxia-inducible factor 1 (HIF-1) (3, 8, 9, 10), the principal transcriptional regulator of the hypoxic response. HIF-1 regulates the expression of genes involved in glycolysis (8, 9), angiogenesis (11), cell survival, and cell cycle control (3, 10). HIF-1 is a heterodimer composed of an oxygen-regulated HIF-1α subunit and a constitutively expressed HIF-1β subunit (12). Under normoxic conditions, HIF-1α is hydroxylated by prolyl hydroxylase domain proteins (13), recognized by the von Hippel-Lindau E3 ligase complex (13), and subsequently degraded by the 26S-proteasome system (13, 14). Oxygen deprivation in hypoxia inhibits prolyl hydroxylase domain activity, stabilizes HIF-1α and promotes HIF-1-dependent transcription through binding of HIF-1 to hypoxia-response elements (HREs) in target gene regulatory regions (13).

While the oxygen-dependent regulation of HIF-1α stability, the characteristics of hypoxic cancer cells and the role of HIF-1 in hypoxic adaptation have been extensively studied, the molecular mechanisms regulating HIF-1 activity remain incompletely understood. In particular, the mechanisms by which HIF-1 promotes adaptive responses associated with reduced proliferation and chemotherapy resistance have not been fully elucidated. Therefore, identification of previously unrecognized regulators of HIF-1 activity may provide important insights into the molecular basis of hypoxic adaptation and reveal novel therapeutic targets for overcoming hypoxia-induced chemoresistance.

We carried out an unbiased shRNA screen to identify novel regulators of HIF-dependent transcription and discovered that debranching RNA lariats 1 (DBR1), a highly conserved lariat RNA debranching enzyme, is a positive regulator of HIF-1 activity. Here, we demonstrate that DBR1 supports HIF-1-dependent transcription and also increases HIF-1 recruitment to HREs in response to hypoxic stimuli. Through shedding light on this function, we establish a noncanonical role of the RNA debranching enzyme DBR1 in hypoxic transcriptional regulation, which is unrelated to its RNA debranching activity. These findings provide a key functional connection between the regulation of RNA metabolism and HIF-1-dependent transcriptional adaptation. Furthermore, DBR1 contributes to hypoxia-induced resistance to conventional chemotherapeutic agents, thereby linking HIF-1-dependent transcriptional adaptation to chemoresistance in hypoxic cancer cells.

## Results

### DBR1 is a novel positive regulator of HIF activity

As a first step toward a genome-wide screen to identify novel positive regulators of HIF-1 activity (Fig. 1*A*), we generated a HIF-responsive reporter cell line by stably introducing an enhanced green fluorescent protein (EGFP) reporter construct driven by five tandem HREs (5HRE) into HeLa cells. Reporter functionality was validated by culturing cells under normoxic or hypoxic conditions. EGFP fluorescence was markedly increased under hypoxia relative to normoxia, confirming that the reporter reflects HIF-dependent transcriptional activity (Fig. 1*B*). Using this system, we performed a loss-of-function screen with a pooled lentiviral shRNA library targeting approximately 15,000 human genes to identify positive regulator of HIF signaling. Following lentivirus transduction and hypoxic exposure, cells exhibiting reduced EGFP fluorescence were enriched by flow cytometric cell sorting (Fig. 1*A*). Eighteen single-cell clones were isolated, and the shRNA sequences integrated into their genomic DNA were identified by Sanger sequencing to nominate candidate positive regulators of HIF-dependent transcription (Fig. 1*A*; Table S1). As a secondary screen, the roles of each candidate in HIF-dependent transcription were evaluated by luciferase reporter assays using the *5HRE-luc* reporter, in which luciferase expression is driven by the HIF-dependent 5HRE promoter. HeLa cells were cotransfected with representative shRNAs identified in the 18 clones, the *5HRE-luc* reporter plasmid, and a Renilla luciferase control plasmid, and cultured under normoxic or hypoxic conditions. Among the candidate genes, *DBR1* knockdown exhibited the most robust and reproducible suppressive effect on the HIF-dependent reporter activity and was therefore selected for further analysis (Fig. 1*C*; Fig. S1). This finding was further confirmed using the HeLa/5HRE-EGFP cells employed in the primary screen (Fig. 1*B*). Notably, genetic manipulation of *DBR1* did not affect basal fluorescence intensity of EGFP, supporting the conclusion that its effect on reporter activity is mediated through HRE-dependent transcription in the first screen (Fig. S2). To further validate the role of DBR1 in HIF-dependent transcription, we performed another luciferase reporter assay and found that DBR1 overexpression significantly increased luminescence under hypoxia (Fig. 1*D*). Consistent with these findings, expression of a representative HIF-1 target gene *MET* positively correlated with DBR1 levels: DBR1 overexpression increased *MET* mRNA (Fig. 1*E*), whereas *DBR1* knockdown decreased its expression (Fig. 1*F*). Moreover, DBR1 overexpression significantly enhanced the hypoxic induction of the HIF-1 target genes *BNIP3*, *ANGPTL4*, and *SLC2A1/GLUT1*, whereas *VEGFA* and carbonic anhydrase 9 showed a trend toward increased expression without reaching statistical significance (Fig. S3). Importantly, DBR1 overexpression also significantly enhanced HIF-dependent reporter activity in primary normal human dermal fibroblast (NHDF) under hypoxic conditions, indicating that the effect of DBR1 on HIF signaling is not restricted to cancer cells (Fig. S4). In NHDF cells, DBR1 overexpression significantly enhanced the hypoxic induction of ANGPTL4, whereas no significant effects were observed for BNIP3, GLUT1, VEGFA, or MET (Fig. S5). Collectively, these results identify DBR1 as a positive regulator of HIF-dependent transcriptional activity.

**Figure 1:**
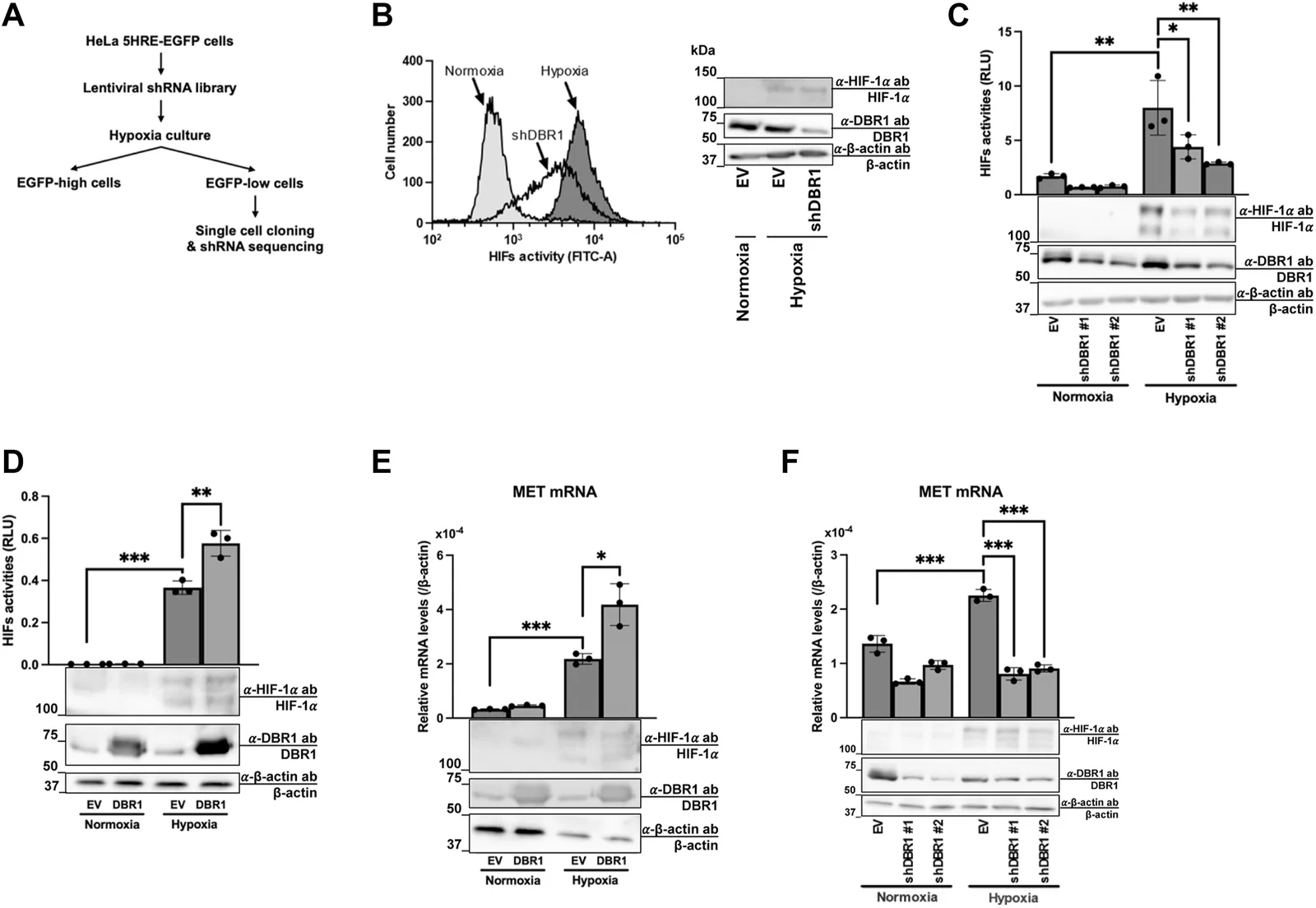
**DBR1 is a novel positive regulator of HIF activity.***A*, schematic overview of the screening strategy used to identify positive regulators of HIF-dependent transcriptional activity. HeLa 5HRE-EGFP cells were transduced with a lentiviral shRNA library and selected with puromycin, followed by exposure to normoxic or hypoxic conditions for 24 h. EGFP-low cells under hypoxia were isolated by FACS sorting (FACSAria II). Single-cell clones were expanded, and integrated shRNAs were identified by Sanger sequencing of their barcode regions. *B*, HeLa 5HRE-EGFP reporter cells were transduced with shDBR1 #1 expression plasmid or its control vector, and cultured with puromycin. Cells were seeded (1 × 10^5^ cells/10-cm dish) and allowed to attach before exposure to normoxic or hypoxic conditions for 24 h. EGFP fluorescence intensity was subsequently analyzed by flow cytometry (FACSCanto II). *C*, *DBR1*-knockdown HeLa cells (1 × 10^4^ cells/well in a 24-well plate) were cotransfected with the p5HRE-luc reporter and a Renilla luciferase control plasmid, followed by exposure to hypoxic conditions for 24 h. HIF-dependent transcriptional activity was assessed by dual-luciferase assay, with firefly luciferase activity normalized to Renilla luciferase activity. HeLa cells transduced with the pRSI9-U6-(sh)-UbiC-TagRFP-2A-Puro plasmid were used as a control (EV). *D*, HeLa cells (1 × 10^4^ cells/well in a 24-well plate) were cotransfected with DBR1 expression plasmid or its empty vector (EV), together with p5HRE-luc and a Renilla luciferase control plasmid. Following exposure to hypoxic conditions for 24 h, luciferase activity was measured, and firefly luciferase activity was normalized to Renilla luciferase activity. *E*, HeLa cells (1 × 10^4^ cells/well in a 24-well plate) were cotransfected with DBR1 expression plasmid or its empty vector (EV) and cultured under normoxic or hypoxic conditions for 24 h before total RNA extraction. *MET* mRNA expression was quantified by qRT-PCR and normalized to β-actin expression. *F*, *DBR1* knockdown HeLa cells (1 × 10^5^ cells/well in a 6-well plate) were cultured under normoxic or hypoxic conditions for 24 h before total RNA extraction. HeLa cells transduced with the pRSI9-U6-(sh)-UbiC-TagRFP-2A-Puro plasmid were used as a control (EV). *MET* mRNA expression was quantified by qRT-PCR and normalized to β-actin expression. Data represent the mean ± SD (n = 3 technical replicates) from a representative experiment (*C*-*F*). Statistical significance was determined using a two-tailed Student’s *t* test. ∗*p* < 0.05, ∗∗*p* < 0.01, ∗∗∗*p* < 0.001. DBR1 overexpression (*D* and *E*) or knockdown (*B*, *C*, and *F*) was confirmed by Western blotting. DBR1, debranching RNA lariats 1; EGFP, enhanced *green* fluorescent protein; FACS, fluorescence-activated cell sorting; HIF, hypoxia-inducible factor; HRE, hypoxia-response element; qRT-PCR, quantitative reverse transcription-polymerase chain reaction.

### DBR1 selectively activates HIF-1- but not HIF-2-dependent transcription

Although these findings indicate that DBR1 enhances HIF-dependent transcription, HRE-based reporter assays do not distinguish between HIF-1 and HIF-2, as both isoforms bind HREs with the shared HIF-1β subunit (15, 16). To determine whether DBR1 preferentially enhances HIF-1- or HIF-2-dependent transcription, we evaluated DBR1-mediated reporter activity in HeLa cells lacking either HIF-1α or HIF-2α. Using the same HIF-responsive luciferase reporter, we evaluated the effect of DBR1 overexpression in each KO background. DBR1 overexpression did not increase luminescence in HIF-1α-deficient cells (Figs. 2*A* and S6*A*), whereas reporter output remained significantly elevated in HIF-2α-deficient cells (Figs. 2*B* and S6*B*). Reexpression of HIF-1α in HIF-1α-KO cells restored DBR1-induced reporter activation (Figs. 2*C* and S6*C*). These results clearly demonstrate that DBR1 promotes HRE-driven transcription in a HIF-1-dependent, but HIF-2-independent, manner.

**Figure 2:**
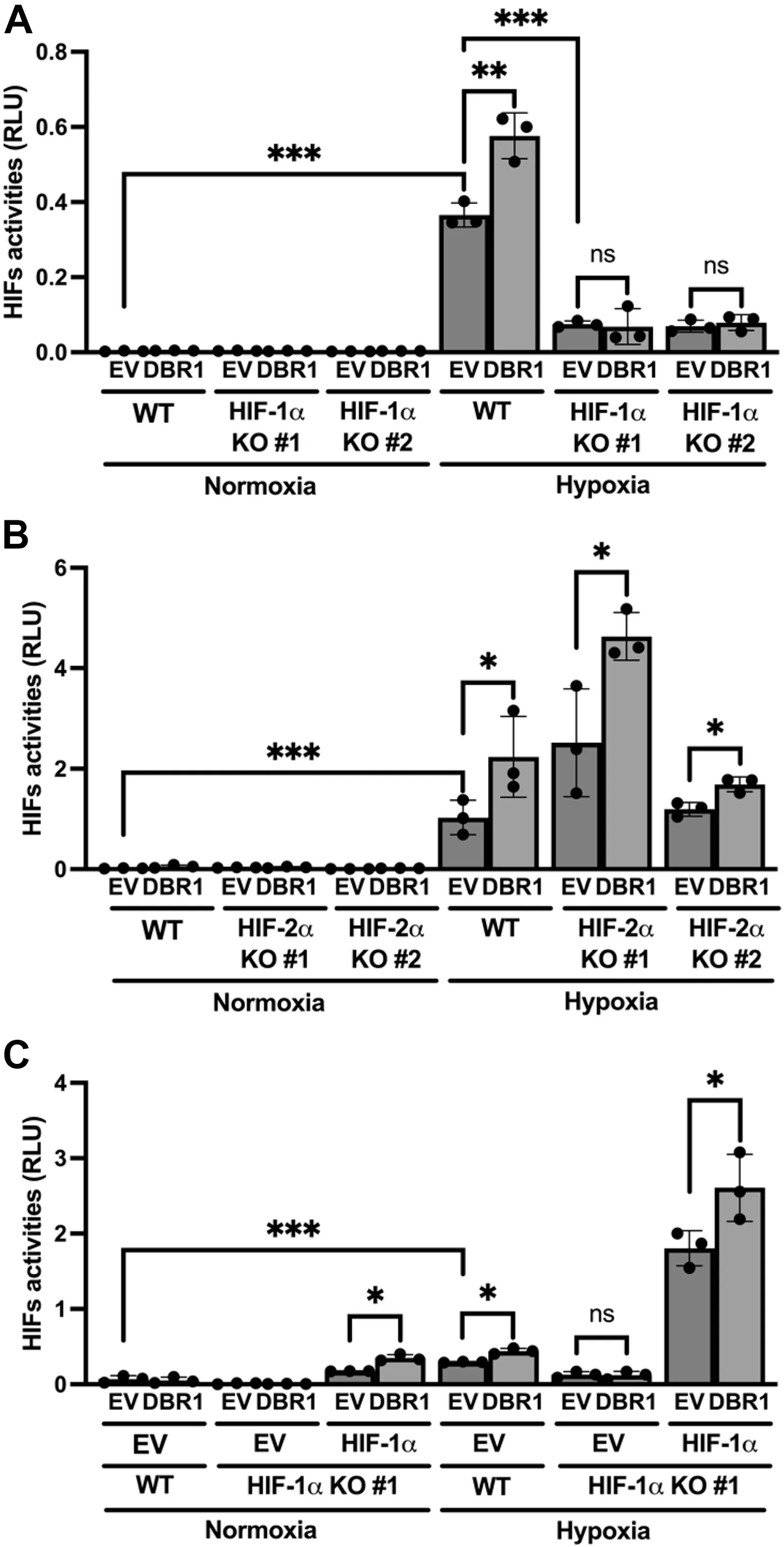
**DBR1 selectively activates HIF-1- but not HIF-2-dependent transcription.***A*, *B*, and *C*, HIF-1α-KO HeLa cells (HIF-1α KO #1 and #2 in *A*) and HIF-2α-KO HeLa cells (HIF-2α KO #1 and #2 in *B*) were seeded in a 24-well plate (1–2 × 10^4^ cells/well) and cotransfected with DBR1 expression plsmid or its empty vector (EV), together with the p5HRE-luc and pRenilla luciferase control plasmids. HeLa cells stably transduced with lentiCRISPRv2 were used as wild-type controls (WT). Twenty-four hours after transfection, cells were cultured under normoxic or hypoxic conditions for an additional 24 h and subjected to dual-luciferase assays. HIF-1α-KO #1 cells were reconstituted with HIF-1α expression vector prior to luciferase analysis (*C*). Data represent the mean ± SD (*n* = 3 technical replicates) from a representative experiment. Statistical significance was determined using a two-tailed Student’s *t* test. ns, not significant, ∗*p* < 0.05, ∗∗*p* < 0.01, ∗∗∗*p* < 0.001. DBR1, debranching RNA lariats 1; HIF, hypoxia-inducible factor.

### DBR1 contributes to HIF-1α nuclear accumulation without altering its mRNA or total protein levels

We sought to identify critical regulatory step through which DBR1 enhances HIF-1 activity. We first examined whether DBR1 upregulates *HIF1A* mRNA expression. Quantitative RT-PCR demonstrated that neither overexpression nor knockdown of DBR1 significantly altered *HIF1A* mRNA levels under hypoxic conditions (Figs. 3, *A* and *B* and S7). We next examined the influence of DBR1 on HIF-1α protein levels because HIF-1α proteolysis is recognized as a major regulatory step of HIF-1 activity (14, 17, 18, 19, 20). Western blotting for HIF-1α protein showed that neither DBR1 overexpression nor *DBR1* knockdown affected HIF-1α protein levels under hypoxia (Fig. 3, *C* and *D*). Together, these results indicate that DBR1 enhances HIF-1 activity through a mechanism independent of *HIF1A* mRNA expression or protein accumulation under hypoxic conditions.

**Figure 3:**
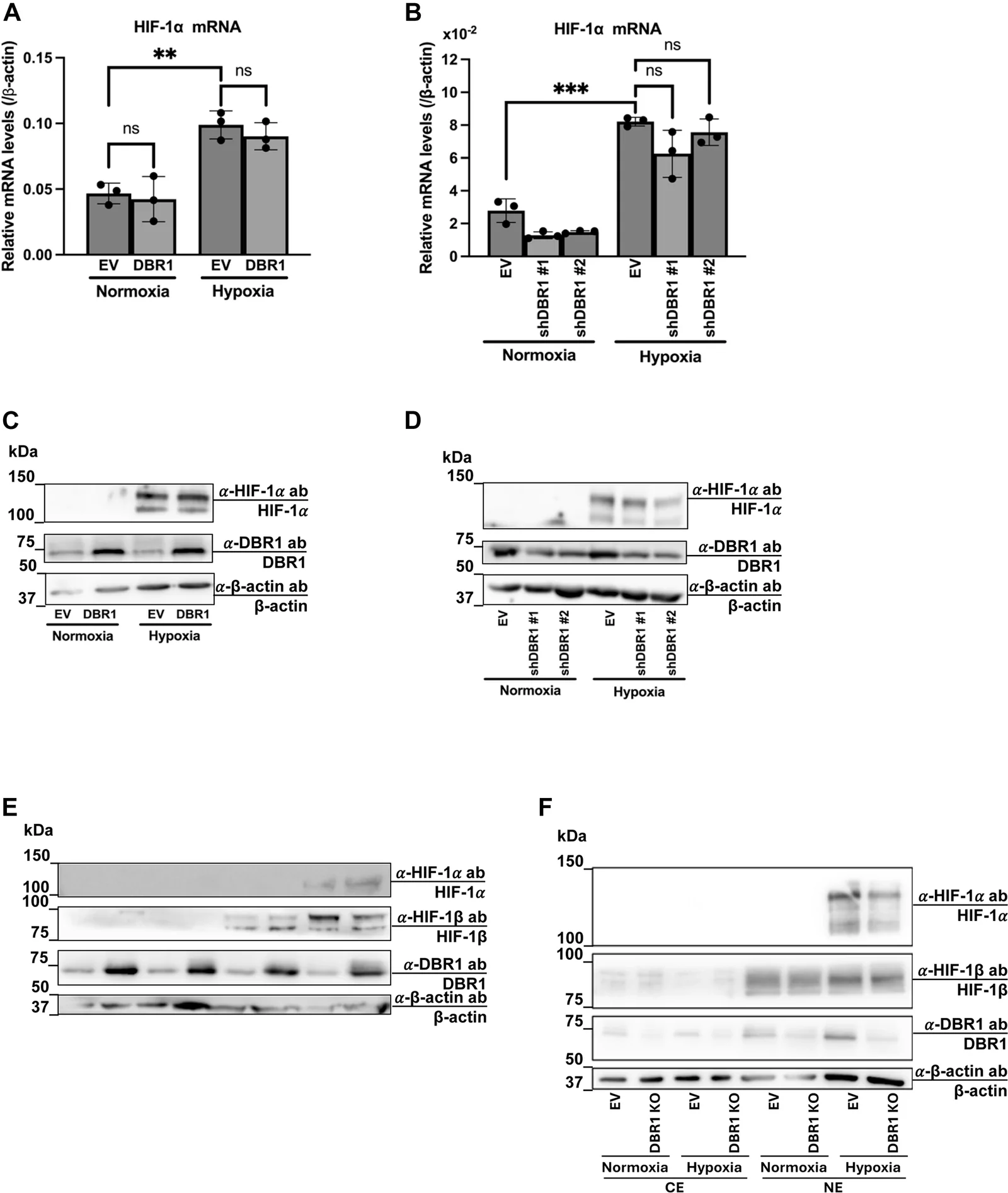
**DBR1 contributes to HIF-1α nuclear accumulation without altering its mRNA or total protein levels.***A* and *C*, HeLa cells (1 × 10^5^ cells/well in a 6-well plate) were transfected with DBR1 expression plasmid or its empty vector (EV). Following exposure to normoxic or hypoxic conditions for 24 h, cells were harvested for analysis of (*A*) *HIF1A* mRNA expression by qRT-PCR, normalized to β-actin mRNA levels, and (*C*) Western blotting using the indicated antibodies. *B* and *D*, *DBR1* knockdown HeLa cells (1 × 10^5^ cells/well in a 6-well plate) were cultured under normoxic or hypoxic conditions for 24 h before extraction of total RNA and proteins. *HIF1A* mRNA expression was quantified by qRT-PCR and normalized to β-actin expression (*B*). Protein extract was subjected to Western blotting using the indicated antibodies (*D*). HeLa cells transduced with the pRSI9-U6-(sh)-UbiC-TagRFP-2A-Puro plasmid were used as a control (EV). *E* and *F*, HeLa cells (1 × 10^5^ cells/well in a 6-well plate) transfected with DBR1 expression plasmid or its empty vector (EV) (*E*) or *DBR1*-KO cells (1 × 10^6^ cells/10-cm dish) (*F*) were cultured under normoxic or hypoxic conditions for 24 h. Cytoplasmic and nuclear extracts (CE and NE, respectively) were prepared and subjected to Western blot analysis to examine protein localization. HeLa cells transduced with lentiCRISPRv2 were used as a control (WT) in *F*. Data represent the mean ± SD (*n* = 3 technical replicates) from a representative experiment (*A* and *B*). Statistical significance was determined using a two-tailed Student’s *t* test. ns, not significant, ∗∗*p* < 0.01, ∗∗∗*p* < 0.001. DBR1, debranching RNA lariats 1; HIF, hypoxia-inducible factor; qRT-PCR, quantitative reverse transcription polymerase chain reaction.

Because HIF-1α functions in the nucleus, its nuclear accumulation is essential for HIF-1-dependent transcription (21). Therefore, we next examined whether DBR1 affects the subcellular localization of HIF-1α. Western blot analysis of cytoplasmic and nuclear fractions revealed that DBR1 overexpression significantly increased nuclear HIF-1α levels, whereas *DBR1* KO significantly reduced them under hypoxic conditions (Fig. 3, *E* and *F*). These results indicate that DBR1 contributes to the nuclear accumulation of HIF-1α without altering its overall abundance.

### DBR1 physically associates with HIF-1α and promotes its recruitment to HREs

Because DBR1 did not affect canonical regulatory steps controlling HIF-1 activity, we next investigated a noncanonical possibility: whether DBR1 directly interacts with HIF-1α to enhance HIF-1 activity. To test this possibility, we performed NanoBiT assays using reciprocal fusion constructs, in which the small and large NanoBiT luciferase fragments were fused to either HIF-1α or DBR1. Physical association between DBR1 and HIF-1α was expected to reconstitute a functional luciferase enzyme and would produce luminescence signals. Compared with the negative control expressing DBR1-LgBiT-N and SmBiT-N, coexpression of DBR1-LgBiT-N with HIF-1α-SmBiT-N significantly increased luminescence (Fig. 4, *A* and *B*). Consistent results were obtained when the NanoBiT fragments were fused to HIF-1α and DBR1 in the reverse configuration. Coexpression of HIF-1α-LgBiT-N and DBR1-SmBiT-N also resulted in increased luminescence, further supporting a physical association between DBR1 and HIF-1α (Fig. 4, *C* and *D*). We further examined the interaction between DBR1 and HIF-1α in primary NHDF using the split luciferase complementation assay. As observed in HeLa cells, increased luminescence indicative of DBR1-HIF-1α interaction was detected in NHDF, showing that this interaction is also detectable in primary human cells. (Fig. S8).

**Figure 4:**
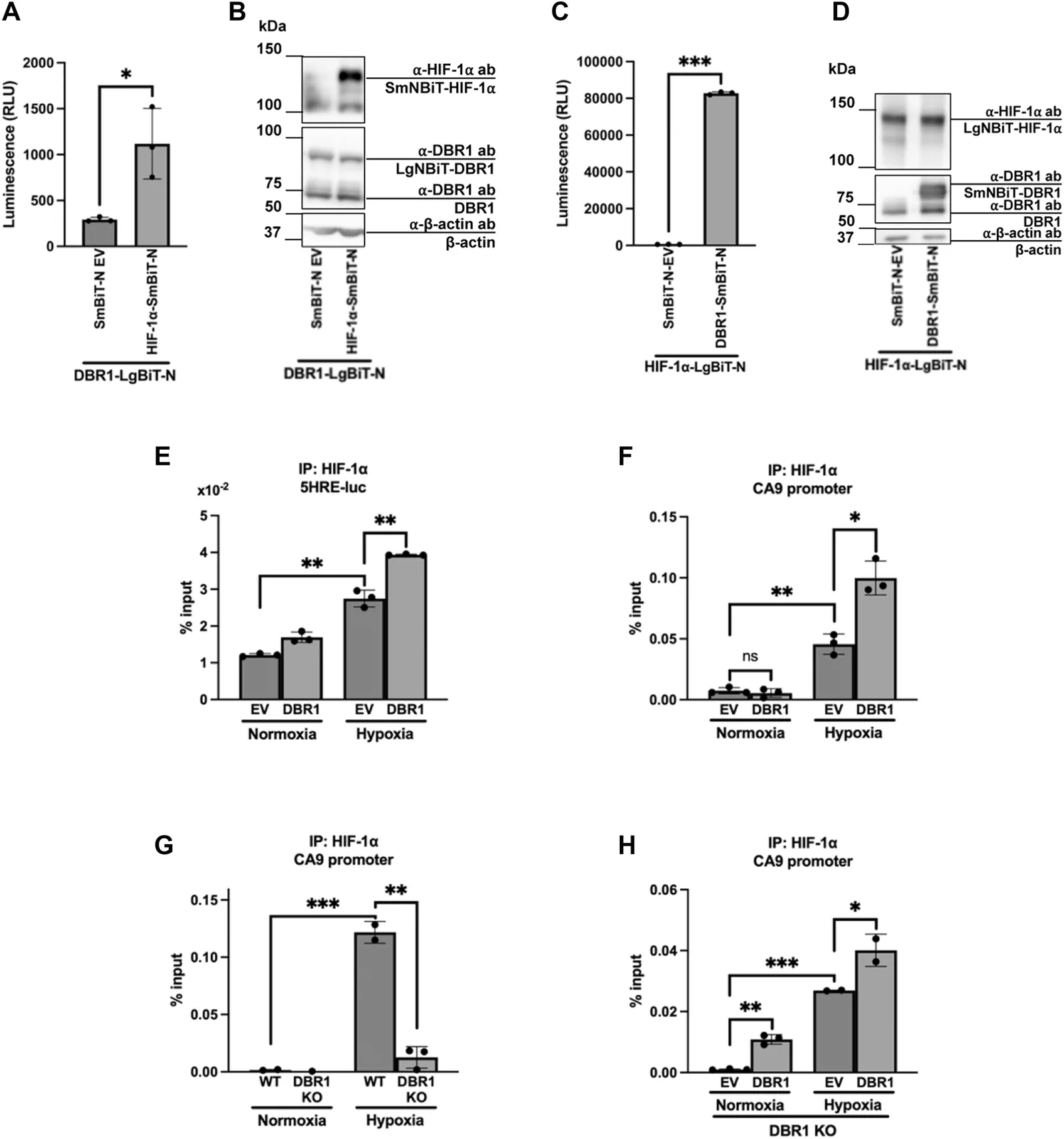
**DBR1 physically associates with HIF-1α and promotes its recruitment to HREs.***A*, *B*, *C*, and *D*, HeLa cells (3 × 10^5^ cells/well in a 6-well plate) were cotransfected with the DBR1-LgBiT-N expression plasmid and the expression plasmid for either HIF-1α-2A-SmBiT-N or SmBiT-N as a negative control (*A* and *B*) or with the HIF-1α-2A-LgBiT-N expression plasmid and the expression plasmid for either DBR1-SmBiT-N or SmBiT-N as a negative control (*C* and *D*). Twenty-four hours after transfection, cells were reseeded (8 × 10^3^ cells/well in a 96-well plate) for NanoBiT analysis (*A* and *C*) or for Western blotting analysis using the indicated antibodies (*B* and *D*). *E*, *F*, *G*, and *H*, After HeLa cells (*E* and *F*), HeLa *DBR1*-KO cells (*DBR1* KO) and control WT cells (HeLa cells transduced with the lentiCRISPRv2) (*G*), and HeLa *DBR1*-KO cells (*H*) were seeded (5 × 10^5^ cells/10-cm dish in *E*, *F*, and *H*; 1 × 10^6^ cells/10-cm dish in *G* and allowed to attach, they were transfected with either DBR1 expression plasmid (DBR1) or its empty vector (EV) (*E*, *F*, and *H*) and with (*E*) or without (*F* and *G*) p5HRE-luc plasmid. Cells were then cultured under normoxic or hypoxic conditions for 24 h and subjected to ChIP using anti-HIF-1α antibody-conjugated beads, followed by qPCR using primers against HRE-containing regions of p5HRE-luc (*E*) or the endogenous CA9 promoter (*F*, *G*, and *H*). Data represent the mean ± SD (*n* = 3 technical replicates) from a representative experiment. Statistical significance was determined using a two-tailed Student’s *t* test. ∗*p* < 0.05, ∗∗*p* < 0.01, ∗∗∗*p* < 0.001. Genetic manipulation of DBR1 in E-H was confirmed by Western blotting, as shown in Figure S9, *A*–*D*, respectively. DBR1, debranching RNA lariats 1; HIF, hypoxia-inducible factor; HRE, hypoxia-response element.

Next, we investigated how the physical interaction between DBR1 and HIF-1α contributes to enhanced HIF-1 activity. We first examined whether this interaction promotes the recruitment of HIF-1 to HREs. Chromatin immunoprecipitation (ChIP) was performed using a reporter-based system to assess HIF-1 binding to HREs. HeLa cells were cotransfected with DBR1 and the p5HRE-luc construct, followed by immunoprecipitation of HIF-1-bound chromatin using an anti-HIF-1α antibody after formaldehyde crosslinking. Quantitative PCR (qPCR) analysis of the HRE sequence within the reporter showed that DBR1 overexpression significantly increased HIF-1 occupancy at the HRE (Figs. 4*E* and S9*A*). To determine whether this effect also operates at endogenous loci, ChIP-qPCR analysis was performed at the HRE-containing promoter of the canonical HIF-1 target gene carbonic anhydrase 9. DBR1 overexpression increased HIF-1 recruitment to this site (Figs. 4*F* and S9*B*), whereas *DBR1* KO reduced occupancy, which was restored upon DBR1 reexpression (Figs. 4, *G*, *H*, S3 and S9*C*, *D*). Collectively, these data demonstrate that DBR1 enhances HIF-1-dependent transcription by promoting HIF-1 recruitment to HREs.

### DBR1 enhances HIF-1-dependent transcription independently of its debranching activity

As a lariat debranching enzyme, DBR1 is required for the turnover of intron lariat RNAs following splicing (22, 23). We therefore tested whether its catalytic activity is required for the enhancement of HIF-1-dependent transcription. We employed a catalytically inactive mutant of DBR1 (H85N&I120T) based on prior studies demonstrating that histidine 85 and isoleucine 120 are highly conserved residues required for catalytic function, and that their substitution abolishes debranching activity (24). Loss of enzymatic activity was assessed by measuring intron lariat RNA accumulation (Fig. 5, *A*–*C*). Because DBR1 hydrolyzes intron lariat RNAs, impaired function is expected to increase their abundance. Lariat levels were quantified by qRT-PCR using primers spanning the branch-point junction, thereby selectively detecting branched intron lariat species but not linear RNA (Fig. 5*A*). Intronic lariats derived from the *ID1* and *DKK1* genes were analyzed as representative substrates (24), and the expected differential effects of WT and mutant DBR1 on lariat accumulation were confirmed (Figs. 5, *B*, *C* and S10*A*). We then assessed the ability of the catalytically inactive mutant to enhance HIF-1-dependent transcription. *DBR1*-KO HeLa cells were complemented with exogenous constructs expressing either WT DBR1 or the catalytically inactive mutant (H85N&I120T) (Figs. 5*D* and S7), and HIF-1 transcriptional output was assessed using the luciferase reporter. Although immunoblotting showed that the mutant was present at lower levels, it enhanced HIF-1 reporter activity to a similar extent as WT DBR1 (Fig. 5, *D* and *E*). These results demonstrate that DBR1 enhances HIF-1-dependent transcription independently of its debranching activity.

**Figure 5:**
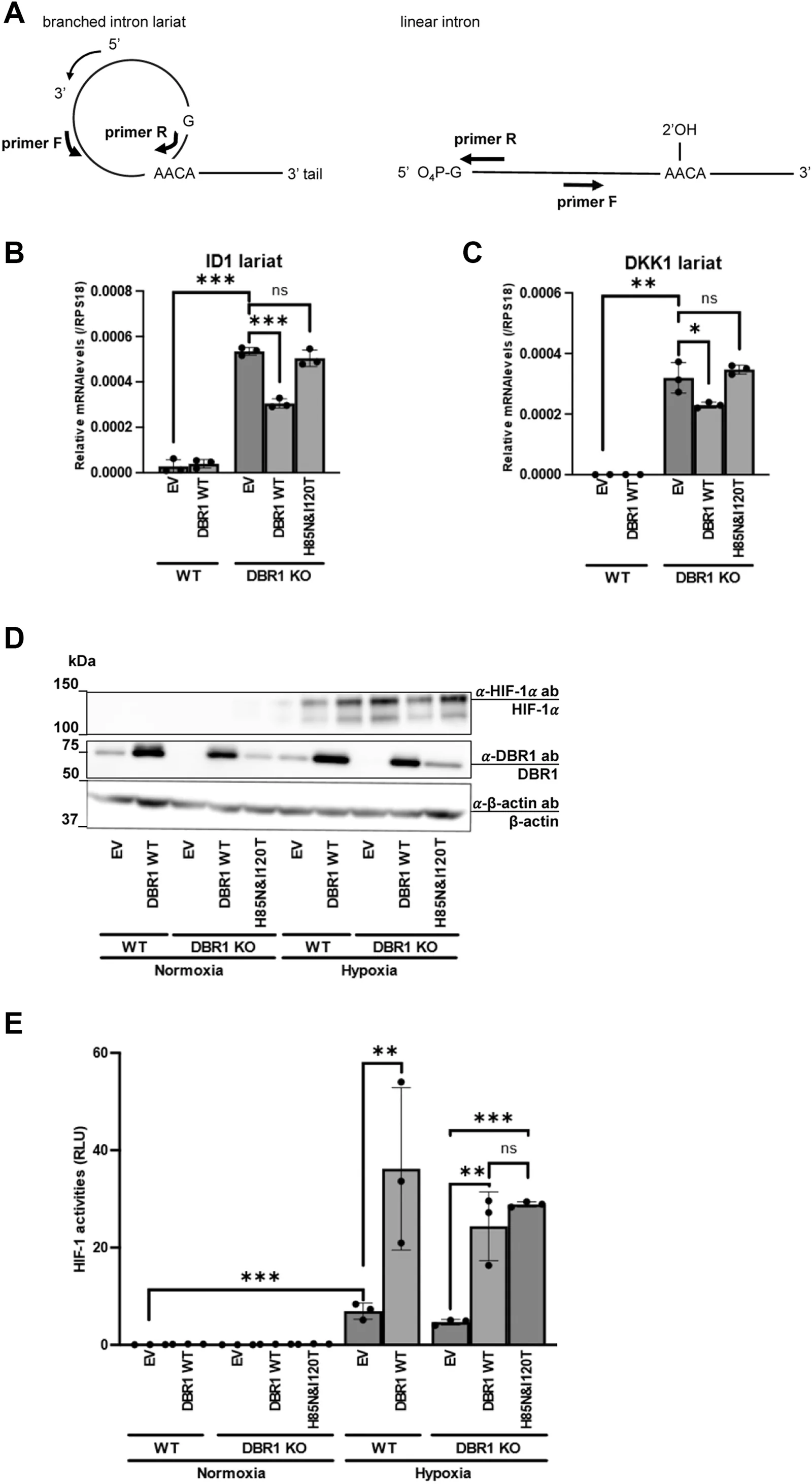
**DBR1 enhances HIF-1-dependent transcription independently of its debranching activity.***A*, schematic illustration of the primer design strategy used to detect branched intron lariats. The reverse primer spans the lariat branch junction and is fully complementary to the sequence created by the 5′ splice site-branchpoint linkage. This sequence is absent in the corresponding linear RNA; consequently, reverse transcription and PCR amplification are specific to the lariat structure and cannot be generated from the linear RNA. *B*, *C*, and *D*, HeLa cells stably transduced with lentiCRISPRv2 (WT) and *DBR1*-KO HeLa cells (2 × 10^5^ cells/well in a 6-well plate in *B* and *C*; 2 × 10^4^ cells/well in a 6-well plate in *D* were stably transduced with the expression vector for either Cas9-resistant DBR1 (DBR1 WT) or DBR1 (H85N&I120T), or their empty vector (EV) as indicated. Total RNA (*B* and *C*) and cell lysates (*D*) harvested from the cells were subjected to qRT-PCR (*B* and *C*) to quantify *ID1* (*B*) and *DKK1* (*C*) intron lariat RNA levels, and to Western blot analysis using the indicated antibodies (*D*). *E*, the same cells as described in *B*-*D* (2 × 10^4^ cells/well in a 24-well plate) were cotransfected with p5HRE-luc and a Renilla luciferase control plasmid, cultured under normoxic or hypoxic conditions for 24 h, and subjected to a dual-luciferase assay to quantify HIF-1 activity. Data represent the mean ± SD (*n* = 3 technical replicates) from a representative experiment (*B*, *C*, and *E*). Statistical significance was determined using a two-tailed Student’s *t* test. ns, not significant, ∗*p* < 0.05, ∗∗*p* < 0.01, ∗∗∗*p* < 0.001. Genetic manipulation of DBR1 in B and C was confirmed by Western blotting, as shown in Figure S10*A*. DBR1, debranching RNA lariats 1; DKK1, Dickkopf WNT signaling pathway inhibitor 1; HIF, hypoxia-inducible factor; ID1, inhibitor of differentiation 1; qRT-PCR, quantitative reverse transcription-polymerase chain reaction.

### Participation of DBR1 in HIF-1-mediated hypoxia signaling promotes lariat RNA accumulation and contributes to impaired cell growth under hypoxia

The noncanonical function of DBR1 to enhance HIF-1 activity appears to operate primarily under hypoxic conditions, when HIF-1 is stabilized and transcriptionally active. To examine whether HIF-1α influences the balance between DBR1’s catalytic (canonical) and noncatalytic (noncanonical) functions and whether lariat RNA accumulates when DBR1 participates in HIF-1 activity enhancement, intron lariat RNA levels were quantified under hypoxia in WT and HIF-1α KO cells. Under these conditions, HIF-1α KO cells exhibited reduced levels of lariat RNA, whereas WT cells showed increased lariat RNA levels (Figs. 6, *A*, *B* and S10*B*). These observations raise the possibility that HIF-1 modulates DBR1 behavior under hypoxia, potentially redirecting it from RNA processing toward the promotion of HIF-1-dependent transcriptional activation. Such altered lariat RNA regulation under hypoxia raises the possibility that lariat RNA accumulation affects cellular physiology. To test this, cell proliferation was assessed in *DBR1*-deficient cells reconstituted with either WT or catalytically inactive DBR1 (H85N&I120T). *DBR1* KO resulted in increased lariat RNA levels and impaired cell growth. Reexpression of WT DBR1 restored lariat RNA levels to baseline, whereas the mutant failed to do so (Figs. 6, *C*, *D* and S10*C*). Notably, the proliferation defect of *DBR1*-KO cells was fully rescued by WT DBR1 reconstitution under normoxic conditions, but only partially rescued under hypoxic conditions (Figs. 6, *E*, *F* and S10*C*), suggesting that hypoxia-associated engagement of DBR1 in HIF-1-dependent transcription limits its availability for lariat RNA processing. These findings support a model in which lariat RNA accumulation resulting from DBR1 participation in HIF-1-mediated hypoxia signaling contributes to impaired cell growth under hypoxic conditions (Fig. 6*G*).

**Figure 6:**
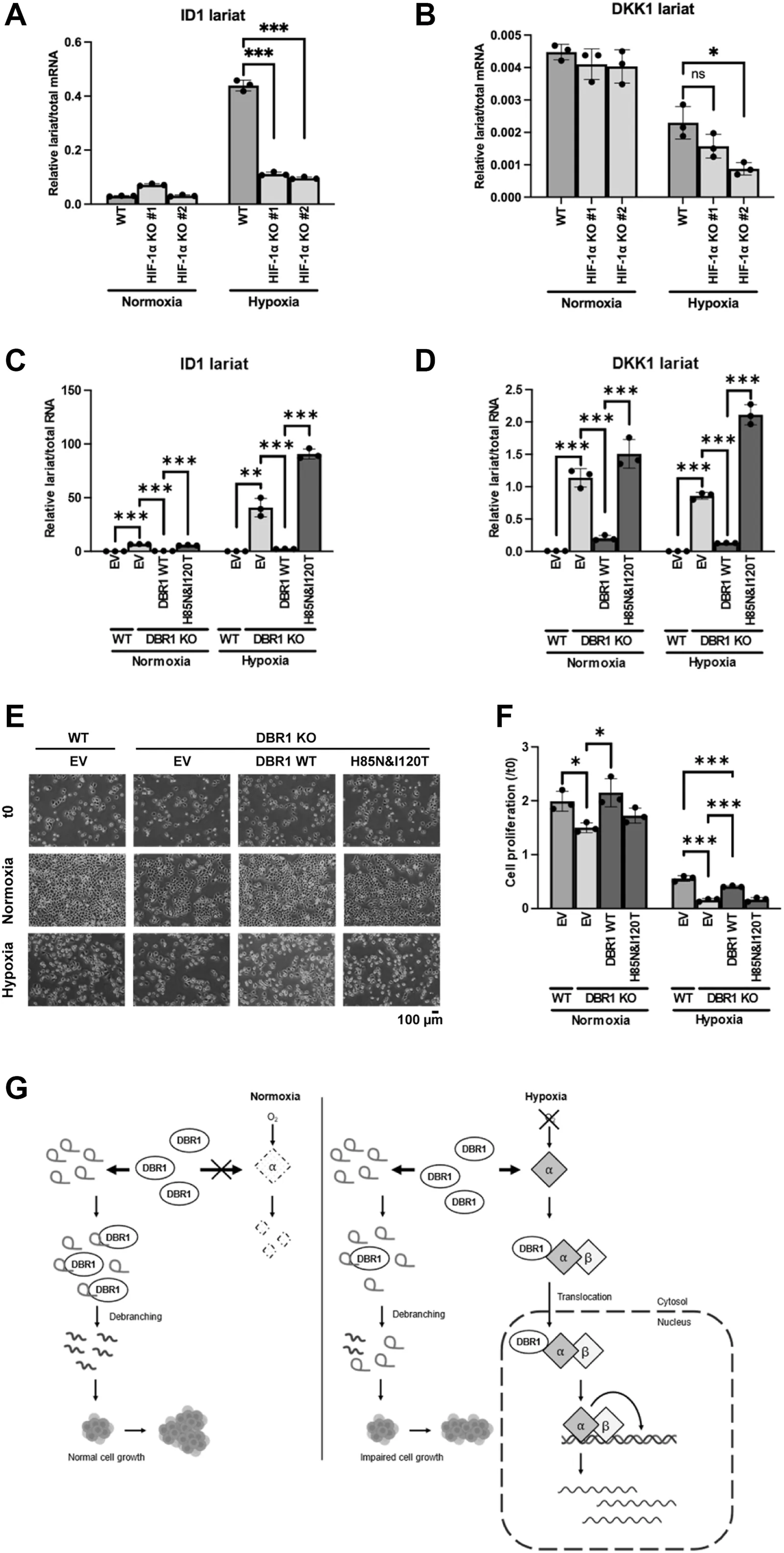
**Participation of DBR1 in HIF-1-mediated hypoxia signaling promotes lariat RNA accumulation and contributes to impaired cell growth under hypoxia.***A* and *B*, HeLa cells stably transduced with lentiCRISPRv2 (WT) and HIF-1α-KO HeLa cells (5 × 10^5^ cells/well in a 6-well plate) were cultured under normoxic or hypoxic conditions for 24 h, and total RNA was subjected to qRT-PCR to quantify *ID1* (*A*) and *DKK1* (*B*) intron lariat RNA levels. *C*, *D*, *E*, and *F*, HeLa cells stably transduced with lentiCRISPRv2 (WT) and *DBR1*-KO HeLa cells (3 × 10^5^ cells/well in a 6-well plate) were stably transduced with the expression vector for either CRISPR/Cas9-resistant DBR1 (DBR1 WT) or DBR1 (H85N&I120T), or their empty vector (EV) as indicated, and cultured under normoxic or hypoxic conditions for 24 h (*C* and *D*) or 36 h (*E* and *F*). Total RNA was extracted to quantify *ID1* (*C*) and *DKK1* (*D*) intron lariat RNA levels by qRT-PCR. Representative images of cell proliferation at the indicated time points from t0 (immediately before normoxic or hypoxic incubation) to 36 h after the incubation (*E*). Quantification of cell proliferation by hemocytometer counting with cell numbers normalized to t0 (*F*). *G*, schematic model illustrating HIF-1-DBR1-dependent regulation of intron lariat RNAs associated with cell proliferation. Data represent the mean ± SD (*n* = 3 technical replicates) from a representative experiment (*A*–*D* and *F*). Statistical significance was determined using a two-tailed Student’s *t* test. ns, not significant, ∗*p* < 0.05, ∗∗*p* < 0.01, ∗∗∗*p* < 0.001. Genetic manipulation of *DBR1* in A–B and C–F was confirmed by Western blotting, as shown in Figure S10, *B* and *C*, respectively. DBR1, debranching RNA lariats 1; DKK1, Dickkopf WNT signaling pathway inhibitor 1; HIF, hypoxia-inducible factor; ID1, inhibitor of differentiation 1; qRT-PCR, quantitative reverse transcription-polymerase chain reaction.

### DBR1 contributes to hypoxia-induced resistance to conventional chemotherapeutic agents

HIF-1 is known to promote resistance of cancer cells to a variety of chemotherapeutic agents through the induction of downstream target genes (3, 8, 9). Therefore, enhancement of HIF-1 activity by DBR1 would be expected to increase chemoresistance under hypoxic conditions. In addition, our findings indicate that participation of DBR1 in HIF-1-mediated hypoxia signaling is associated with lariat RNA accumulation and impaired cell growth under hypoxia. Because conventional chemotherapeutic agents preferentially target actively proliferating cancer cells, we reasoned that growth retardation associated with lariat RNA accumulation might further contribute to chemoresistance. We therefore examined whether DBR1 affects the sensitivity of cancer cells to conventional chemotherapeutic agents under hypoxic conditions. HeLa WT and *DBR1*-KO cells were treated with representative conventional chemotherapeutic agents, including camptothecin and carboplatin, under hypoxic conditions, and their effects on cell growth were evaluated. Hypoxia significantly increased cell survival following treatment with each chemotherapeutic agent in WT cells, but not in *DBR1*-KO cells. (Fig. 7, *A* and *B*). These results indicate that DBR1 contributes, at least in part, to hypoxia-induced resistance to conventional chemotherapeutic agents.

**Figure 7:**
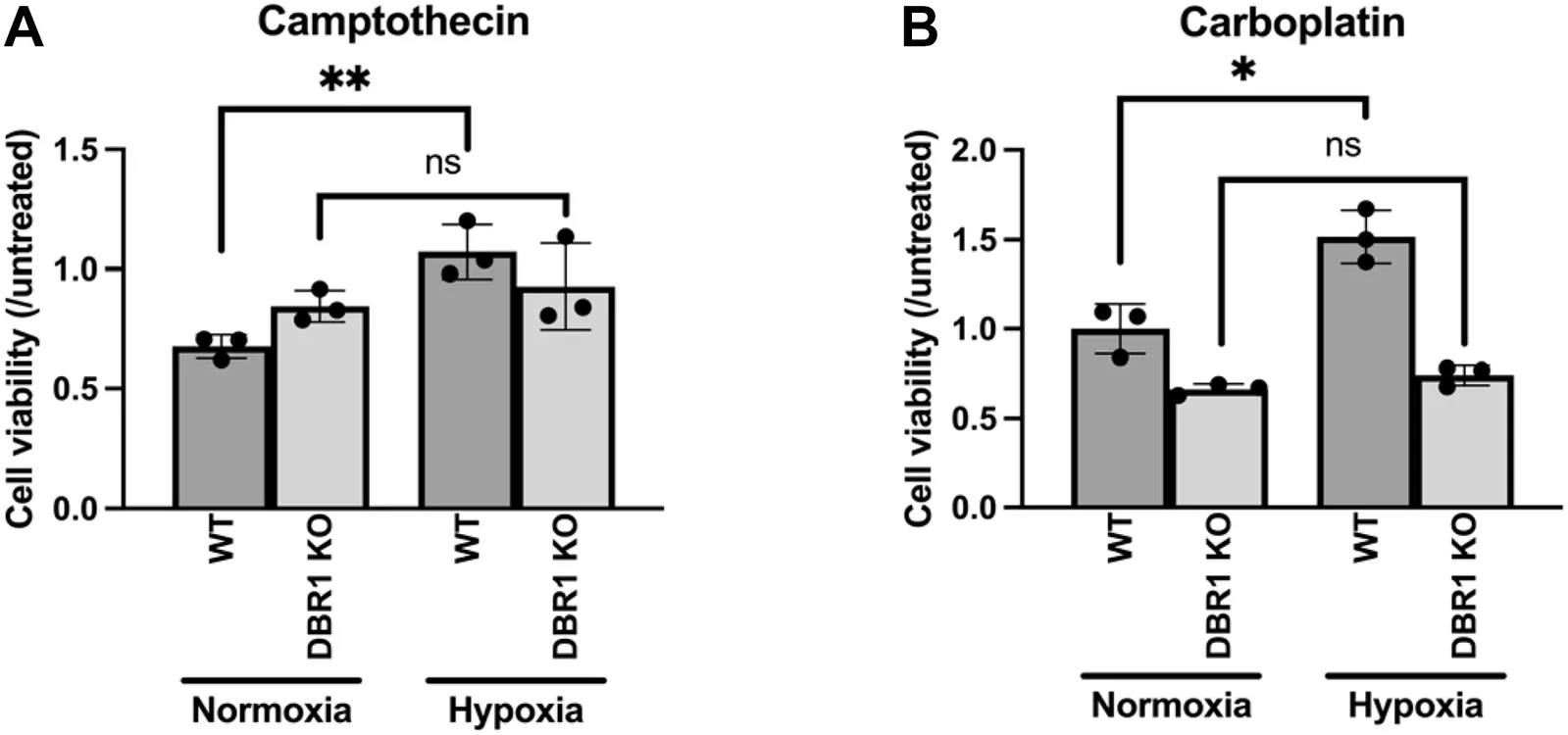
**DBR1 contributes to hypoxia-induced resistance to conventional chemotherapeutic agents.***DBR1*-KO HeLa cells (1 × 10^3^ cells/well in a 96-well plate) were seeded overnight and cultured under normoxic or hypoxic conditions for 24 h. Cells were treated with the camptothecin (*A*) or Carboplatin (*B*) for an additional 24 h. Cell viability was assessed using the colorimetric Cell Count Reagent SF. Viability was normalized to that of untreated cells cultured under the corresponding oxygen conditions. Data represent the mean ± SD (*n* = 3 technical replicates) from a representative experiment. Statistical significance was determined using a two-tailed Student’s *t* test. ns, not significant, ∗*p <* 0.05, ∗∗*p* < 0.01, ∗∗∗*p* < 0.001. DBR1, debranching RNA lariats 1.

## Discussion

In the present study, we identified DBR1 as a previously unrecognized positive regulator of HIF-1. DBR1 physically associated with HIF-1α, promoted HIF-1 recruitment to HREs, and enhanced HIF-1-dependent transcription independently of its canonical lariat RNA debranching activity. Under hypoxic conditions, HIF-1 appeared to functionally redirect DBR1 from lariat RNA processing toward HIF-1-dependent transcriptional regulation, resulting in lariat RNA accumulation and impaired cell proliferation. Furthermore, DBR1 contributed to hypoxia-induced resistance to multiple conventional chemotherapeutic agents, possibly by promoting a growth-retarded state that reduces the susceptibility of cancer cells to agents targeting actively proliferating cells, supporting a model in which redistribution of DBR1 function promotes hypoxic adaptation and chemoresistance at the expense of lariat RNA processing.

Because both HIF-1 and HIF-2 recognize HREs, we examined the specificity of DBR1-mediated regulation by employing selective KO of either HIF-1α or HIF-2α while preserving the shared HIF-1β (ARNT) subunit. DBR1-dependent reporter activation was abolished in HIF-1α-KO cells, but retained in HIF-2α-KO cells, and restored following HIF-1α reexpression, indicating selective regulation of HIF-1-dependent transcriptional responses. Although HIF-1 and HIF-2 share similar HRE motifs, they operate through distinct cofactor dependencies and transcriptional outputs (15, 16), raising the possibility that DBR1 preferentially participates in HIF-1-associated transcriptional complexes.

DBR1 is best known as a lariat RNA debranching enzyme that catalyzes intron lariat processing during pre-mRNA splicing (22, 23). However, our finding that DBR1 enhanced HIF-1-dependent transcription independently of its debranching activity suggests a previously unrecognized noncanonical function of this protein. Recent studies have shown that several RNA-processing factors can also participate in transcriptional and chromatin-associated regulatory pathways (25, 26, 27). Our data extend this concept by identifying DBR1 as a potential link between lariat RNA metabolism and hypoxia-dependent transcriptional regulation. These findings further raise the possibility that DBR1 functions as a moonlighting protein with separable roles in RNA metabolism and transcriptional regulation.

Mechanistically, DBR1 did not alter the abundance of *HIF1A* mRNA or total protein levels but contributed to the nuclear accumulation of HIF-1α under hypoxic conditions. In addition, ChIP-qPCR analysis demonstrated that DBR1 enhances HIF-1 occupancy at HRE-containing target loci. These findings suggest that DBR1 regulates HIF-1 signaling at multiple steps downstream of HIF-1α stabilization, contributing to its nuclear accumulation and facilitating the efficient recruitment or stabilization of HIF-1 on chromatin, thereby promoting hypoxia-responsive transcription through a mechanism independently of its canonical role in RNA lariat processing.

Interestingly, the enhancement of HIF-1 activity by DBR1 did not uniformly increase the expression of all HIF-1 target genes examined. In HeLa cells, DBR1 overexpression significantly enhanced the hypoxic induction of MET, BNIP3, ANGPTL4, and GLUT1, whereas in NHDF cells, among the HIF-1 target genes examined, a significant effect was observed only for ANGPTL4. We have previously shown that the subset of HIF-1 target genes preferentially induced under hypoxic conditions differs depending on both the upstream factor that enhances HIF-1 activity and the cellular context [32]. Consistent with this concept, our present findings suggest that DBR1 preferentially enhances the hypoxic induction of specific subsets of HIF-1 target genes in a cell type-dependent manner. Such target selectivity may reflect differences in the transcriptional regulatory context of individual HIF-1 target genes, including the availability of transcriptional cofactors and chromatin accessibility. Further studies will be required to determine the molecular basis by which DBR1 selectively influences HIF-1 transcriptional output in different cellular contexts.

Hypoxic stress has increasingly been associated with widespread remodeling of RNA metabolism, including altered splicing dynamics and stress-responsive RNA processing pathways (7, 28). Quantification of intron lariat RNA suggested a functional interplay between the canonical RNA debranching activity of DBR1 and its noncanonical role in HIF-1 regulation. Under hypoxic conditions, HIF-1α-deficient cells exhibited reduced lariat accumulation, consistent with preserved DBR1 debranching activity in the absence of HIF-1 engagement. In comparison, WT cells displayed increased lariat RNA accumulation during hypoxia, suggesting that participation of DBR1 in HIF-1-dependent transcription partially limits its availability for RNA lariat processing. Together, these observations support a model in which DBR1 functionally shifts between RNA-processing and transcription-associated functions during hypoxic adaptation. Given the pervasive coupling between transcription and RNA processing (29), the dual functionality of DBR1 may reflect dynamic coordination between RNA metabolism and hypoxia-responsive transcriptional programs. Additional support for this model comes from the observation that cell proliferation was only partially restored by WT DBR1 under hypoxic conditions. Although reconstitution of WT DBR1 restored lariat RNA debranching activity, such restoration failed to fully normalize cell growth under hypoxia, suggesting that engagement of DBR1 in HIF-1-dependent transcription may reduce its availability for RNA lariat processing. These findings are consistent with a model in which hypoxia-driven redistribution of DBR1 function contributes to lariat RNA accumulation and impaired cell proliferation. Whether the accumulated lariat RNAs themselves have functional consequences under hypoxic conditions remains an important question for future investigation.

In addition to its role in hypoxic adaptation, DBR1 contributed to resistance against multiple conventional chemotherapeutic agents under hypoxic conditions. HIF-1 is known to promote chemoresistance through the induction of downstream genes involved in cell survival, metabolic adaptation, and stress responses. Therefore, the ability of DBR1 to enhance HIF-1 activity likely contributes, at least in part, to hypoxia-induced chemoresistance. Our findings further suggest that lariat RNA accumulation and the associated proliferation defect may represent an additional mechanism contributing to hypoxia-induced chemoresistance, particularly against agents that preferentially target actively proliferating cells. Future studies will be required to determine the relative contribution of HIF-1 activation and altered lariat RNA metabolism to DBR1-mediated chemoresistance.

Several limitations of this study should be acknowledged. Although endogenous HIF-1 target genes were also examined, HIF-1 activity was primarily evaluated using reporter-based assays, which may not fully recapitulate endogenous chromatin and transcriptional regulation. Second, although DBR1-mediated enhancement of HIF-1 activity was observed in both HeLa and NHDF cells, the mechanistic studies were conducted predominantly in HeLa cells. Further investigation in additional cell types will therefore be required to determine the extent to which the molecular mechanisms and target-gene selectivity of DBR1-mediated HIF-1 regulation are conserved across different cellular contexts. Finally, the broader physiological and pathological implications of DBR1-mediated HIF-1 regulation warrant further investigation.

Our study reveals that DBR1 is an uncharacterized positive regulator of HIF-1-dependent transcription and highlights a noncanonical function for the RNA debranching enzyme in hypoxic signaling. Collectively, these findings indicate that DBR1 couples RNA lariat metabolism and HIF-1-dependent transcription during cellular adaptation to hypoxia. Our findings support a functional redistribution model of DBR1 in which hypoxia-stabilized HIF-1 engages DBR1 as a coregulator of HIF-1-dependent transcription. This engagement may reduce the availability of DBR1 for lariat RNA processing, resulting in lariat RNA accumulation and altered cell proliferation, which may in turn contribute to hypoxic chemoresistance.

Because DBR1 contributes to HIF-1 activation, hypoxic adaptation, and resistance to conventional chemotherapeutic agents, disruption of DBR1 function or of the DBR1-HIF-1 interaction may represent a potential therapeutic strategy for targeting hypoxic tumors. However, given the importance of the canonical RNA-processing function of DBR1, the feasibility and safety of such approaches will require careful evaluation. Together, these findings identify DBR1 as a previously unrecognized component of hypoxic adaptation and suggest that targeting DBR1-mediated hypoxia signaling may provide new opportunities for the treatment of hypoxic tumors.

## Experimental procedures

### Cell culture

HeLa and HEK293TN cells were obtained from the American Type Culture Collection and maintained in Dulbecco's Modified Eagle Medium (DMEM) (Nacalai Tesque, Cat. #16971-55) supplemented with 5% fetal bovine serum (Biosera, Cat. #FB-1003) and 1% penicillin-streptomycin (Nacalai Tesque, Cat. #09366-44) at 37 °C in a humidified incubator with 95% air and 5% CO_2_. NHDF cells were purchased from KURABO (Cat. #KF-4009) and maintained in FibroLife Comp kit (KURABO, Cat. #LFC-LL0001). For hypoxic treatments, cells were incubated in a RUSKINN INVIVO2 500 Hypoxic Workstation (Ruskin Technologies) under < 0.1% O_2_, 5% CO_2_, and a balance gas consisting of 5% H_2_ and 95% N_2_ for the indicated durations.

### Plasmids

The pCDH/5HRE-EGFP plasmid was generated by modifying the pCDH/EF1α-MCS-IRES-Puro vector (System Biosciences, Cat. #CD532A-2). The DNA fragment encoding EGFP was first amplified from pEGFP-C1 (Clontech, Cat. #6084-1) using the forward primer: 5′-CACCATGGTGAGCAAGGGCGAGGAGC-3′ and the reverse primer: 5′-TGTCTAGATTACTTGTACAGCTCGTCCATG-3′, digested with NcoI and XbaI, and subsequently inserted downstream of the 5HRE sequence in the p5HRE-luc plasmid (30) using the corresponding restriction sites. The DNA fragment for the 5HRE-EGFP sequence was amplified using the forward primer: 5′-ATATCGATACCGAGCTTTTCTCGAGCCAC-3′ and the reverse primer: 5′-GGGTCGACTTACTTGTACAGCTCGTCCATG-3′, followed by digestion with ClaI and SalI. The pCDH backbone was digested with the same enzymes to remove the EF1α-MCS-IRES-puromycin resistance cassette, and the digested 5HRE-EGFP fragment was ligated into the corresponding restriction sites.

The DNA fragment encoding human DBR1 was amplified from HeLa cell cDNA by nested PCR. The first-round PCR was performed using the forward primer: 5′-CCAGGCGGTGGCTATACTTATT-3′ and the reverse primer: 5′-AGTCCTTGGAGGCAAAAATCCT-3′. The second-round PCR was carried out using the forward primer: 5′-CGATTCTAGAGCCACCATGCGGGTGGCTGTGG-3′ and the reverse primer: 5′- TAAGCGGCCGCCTAAGCTGCATCGTC -3′. The amplified DNA fragment was digested with XbaI and NotI and cloned into the pCDH/EF1-MCS-IRES-Puro vector using the corresponding restriction sites to generate pCDH/DBR1-Puro.

To construct pCDH/EF1-MCS-IRES-Neo, DNA fragment encoded neomycin resistance gene was amplified from pcDNA3.1A (Thermo Fisher Scientific) using the forward primer: 5′-AAGATATCACCATGATTGAACAAGATGG-3′ and the reverse primer: 5′-AAGTCGACTCAGAAGAACTCGTCAAGAAGG-3′. The amplified fragment was digested with EcoRV and SalI. The pCDH backbone was digested with MscI and SalI to remove the puromycin resistance cassette, and the DNA fragment encoding the neomycin resistance gene was ligated into the corresponding blunt and cohesive ends. pCDH/DBR1-Neo was constructed using similar strategy with pCDH/DBR1-Puro by inserting the XbaI and NotI digested DBR1 fragment to pCDH/EF1-MCS-IRES-Neo. A Cas9-resistant pCDH/DBR1-Neo construct, designated pCDH/DBR1-Neo (Cas9-resistant), was generated by introducing silent nucleotide substitutions that did not alter the amino acid sequence at residues 32 and 47 *via* site-directed mutagenesis using the following primers: the forward primer: 5′-CGGCGCGGCCCGGGGCCTGTtGACCTCTTGCTGTGCTGCGG-3′ and the reverse primer: 5′-GCCATGCAGCGTAGATCCGCtTCGTTGCGCACCGCCTGGAA-3′ (lowercase letters indicate mutated nucleotides).

The pCDH-DBR1 (H85N & I120T)-Neo, which expresses a catalytically impaired DBR1 mutant, was generated from the Cas9-resistant pCDH-DBR1-Neo plasmid by introducing two amino acid substitutions (H85N and I120T) *via* site-directed mutagenesis using the following primers: the forward primer: 5′-TTGGGGGAAACAACGAAGCCTCAAATCATT-3′ and the reverse primer: 5′-AGGCTTCGTTGTTTCCCCCAATGAAGAGCG-3′ for H85N; and the forward primer; 5′-GTGTAAGGACCGGTGGAATCTCTGGTATCT-3′ and the reverse primer: 5′-ATTCCACCGGTCCTTACACCTCGGTATTTT-3′ for I120T.

The pCDH/HIF-1α was constructed as described previously (31).

pcDNA4/HIF-1α-2A-LgBiT-N was constructed as previously described (32). pcDNA4/HIF-1α-2A-SmBiT-N was constructed using a similar strategy to that used for pcDNA4/HIF-1α-2A-LgBiT-N. The DNA fragment encoding SmBiT was amplified from pBiT2.1-N [TK/SmBiT] (Promega), and the DNA fragment encoding HIF-1α-2A was amplified from pcDNA4/HIF-1α-2A (33).

To construct pcDNA4/EV-LgBiT-N and pcDNA4/EV-SmBiT-N, DNA fragments encoding LgBiT and SmBiT were amplified from pBiT1.1-N [TK/LgBiT] (Promega) and pBiT2.1-N [TK/SmBiT], respectively, using the forward primer: 5′-CGAGCGACCCTGCAGCGACCCGCTTAAAAG-3′ and the reverse primer: 5′-GACTGGATCCGCCACCACCGGAACTCCC-3′. The amplified fragments were digested with HindIII and BamHI and inserted into pcDNA4/myc-His A (Thermo Fisher Scientific) using the corresponding restriction sites.

To construct pcDNA4A/DBR1-LgBiT-N, the DNA fragment encoding DBR1 was amplified from pCDH/DBR1-Puro using the forward primer: 5′-ATATGGATCCGCCACCATGCGGGTGGCTGTGG-3′ and the reverse primer: 5′-TAAGCGGCCGCCTAAGCTGCATCGTC-3′. The amplified fragment was digested with BamHI and NotI. The previously prepared LgBiT fragment and pcDNA4A backbone were digested with HindIII/BamHI and HindIII/NotI, respectively, and subjected to a three-piece ligation reaction to generate pcDNA4A/DBR1-LgBiT-N.

To construct pcDNA4A/DBR1-myc-SmBiT-N, the DNA fragment encoding DBR1 was amplified from pCDH/DBR1-Puro using the forward primer: 5′-ATATGGATCCGCCACCATGCGGGTGGCTGTGG-3′ and the reverse primer: 5′-TGCAGGATCCAGCTGCATCGTCATCATCA-3′. The amplified fragment was digested with BamHI and inserted into pcDNA4A/EV-SmBiT-N at the BamHI site.

Annealed oligonucleotides encoding shRNA sequences targeting *DBR1* (#1: 5′-CCTGTCTTGGCCTTCTAAAGT-3′ and #2: 5′-GCAAACCTTCTACAGGTATTA-3′) were cloned into the BbsI site of the pRSI9-U6-(sh)-UbiC-TagRFP-2A-Puro vector (Addgene, Cat. #DVLIB-PS) to generate shDBR1 #1 and shDBR1 #2 plasmids. The shRNA plasmids used for candidate screening and validation were generated similarly, using the targeting sequences listed in Table S2.

To construct lentiCRISPRv2-DBR1-puro and lentiCRISPRv2-DBR1-bsd, we utilized the original lentiCRISPRv2 plasmid (Addgene, Cat. # 52961) and a modified version carrying blasticidin resistant gene from an intermediate plasmid px500/cas9-T2A-bsd. To construct px500/cas9-T2A-bsd, the T2A fragment was amplified from px458 (Addgene, Cat. #48138) using the forward primer F1: 5′-AAGGAATTCGGCAGTGGAGAGGG-3′ and the reverse primer R1: 5′-GACAAAGGCTTGGCTGGGCCAGGATTCTCC-3′. The fragment encoding blasticidin resistance gene was amplified from pEF6B-ZBTB2 (34) using the forward primer F2: 5′-AGAATCCTGGCCCAGCCAAGCCTTTGTCTC-3′ and the reverse primer R2: 5′-TTAGAATTCGCCCTCCCACACATAACC-3′. The two fragments were fused by overlap extension PCR using F1 and R2 primers to generate the T2A-BSD fragment. The BbsI restriction site within the coding sequence of the blasticidin resistance gene was disrupted without altering the amino acid sequence by site-directed mutagenesis using the following primers: the forward primer: 5′-CCCATCTCTGAgGACTACAGCGTCGCCAGC-3′ and the reverse primer: 5′-GACGCTGTAGTCcTCAGAGATGGGGATGCTG-3′ (lowercase letters indicate mutated nucleotides). The resulting fragment was digested with EcoRI. The px458 backbone was digested with EcoRI to remove the T2A-GFP cassette, and the T2A-BSD fragment was ligated into the corresponding restriction site.

To construct lentiCRISPR-v2-bsd, the blasticidin resistance gene fragment and T2A fragment were amplified from px500/Cas9-T2A-bsd using the following primer pairs, respectively: bsd forward primer: 5′-AAAGGATCCGCCAAGCCTTTGTCTCAAGA-3′ and the reverse primer: 5′-AAAACGCGTGCCCTCCCACACATAACCAG-3′; and T2A forward primer: 5′-AAAAGATCTGAGGGCAGAGGAAGTCTG-3′ and the reverse primer: 5′-AAAAGATCTTGGGCCAGGATTCTCCTCG-3′. The bsd fragment was digested with BamHI and MluI. The lentiCRISPR-v2 backbone was digested with BamHI and MluI to remove the puromycin resistance cassette, and the digested bsd fragment was ligated into the corresponding restriction sites. The T2A fragment was digested with BglII and inserted into the resulting plasmid at the corresponding restriction site to generate lentiCRISPR-v2-bsd.

The sgRNA targeting the coding sequence of *DBR1* (sgRNA #1: 5′-CAGCACAGCAAGAGGTCGAC-3′ and sgRNA #2: 5′-CTTCCAGGCGGTGCGCAACG-3′) were designed by CRISPRdirect (https://crispr.dbcls.jp/). Oligonucleotides encoding the sgRNA cloned into the BsmBI recognition site of the lentiCRISPRv2 and the lentiCRISPRv2-bsd to construct lentiCRISPRv2-DBR1-puro and lentiCRISPRv2-DBR1-bsd, respectively.

### Lentiviral production and generation of stable cell lines

HEK293TN cells were seeded in antibiotic-free DMEM supplemented with 5% fetal bovine serum and transfected with pPACK HI-GAG, pPACK HI-REV, pVSV-G (System Biosciences), and plasmids of interest using PEI MAX in serum- and antibiotic-free DMEM. Medium was replaced after 16 h, and lentivirus-containing supernatant was collected 48 h posttransfection and filtered through a 0.45 μm filter (Merck Millex GS). Target cells were seeded prior to infection and treated with polybrene (16 μg/ml) for 30 min. Lentivirus-containing medium was then added, and cells were maintained under standard culture conditions for 2 days prior to subsequent treatments. Lentiviral particles produced from the pCDH/5HRE-EGFP plasmid were used to infect HeLa cells. Single-cell clones were isolated and expanded to establish the HeLa/5HRE-EGFP cell line for subsequent experiments. Stable cell lines reconstituted with DBR1 or DBR1 (H85N & I120T) were generated by lentiviral transduction using pCDH/DBR1-Neo (Cas9-resistant) or pCDH/DBR1 (H85N & I120T)-Neo, respectively. Cells transduced with the pCDH-Neo empty vector were used as controls. All stable cell lines were selected with the corresponding antibiotics.

### shRNA library screening and generation of DBR1 knockdown cells

For shRNA library screening, lentivirus was produced and HeLa/5HRE-EGFP cells were transduced using the same protocol described previously. Cells transduced with Cellecta Decipher shRNA library (Cat. #DHPAC-M1-P, DHDAC-M2-P, and DHCSC-M3-P) were selected with puromycin (Fujifilm, Cat. #160-23151) (2 μg/ml) for 5 days. Sorted cells were recovered and expanded as single-cell colonies. Genomic DNA was extracted from individual colonies using the MagExtractor Genome High Efficient Genomic DNA Purification Kit (Toyobo, Cat. #NPK-101). The integrated shRNAs were identified by sequencing their unique barcode regions and matching the barcode sequences to the corresponding shRNA targets according to the manufacturer’s annotation file. *DBR1* knockdown HeLa cell lines were generated by lentiviral transduction using shRNA expression plasmids shDBR1 #1 and shDBR1 #2. The HeLa cell lines with stable knockdown of candidate genes for screening and validation were generated similarly, using the shRNA sequences listed in Table S2.

### Establishment of KO cells

*HIF1A*- and *EPAS1*-KO HeLa cells were generated using lentiviral CRISPR/Cas9 systems as described previously (31). *DBR1*-KO HeLa cells were generated sequential lentiviral transduction using lentiviruses produced from lentiCRISPRv2-DBR1-puro and lentiCRISPRv2-DBR1-bsd, which express sgRNA targeting *DBR1*. Each plasmid was first cotransfected with the pPACK lentiviral packaging system to produce lentiviral particles. HeLa cells were sequentially transduced with the corresponding lentiviruses and subsequently selected with puromycin and blasticidin (Funakoshi, Cat. #KK-400). Single-cell clones were isolated and expanded. Successful *DBR1* KO was confirmed by genomic DNA sequencing and Western blot analysis prior to use in subsequent experiments. Cells transduced with the lentiCRISPRv2 empty vector were used as controls.

### Plasmid transfection

Cells were seeded and allowed to attach to the culture plates overnight. Plasmid DNA was mixed with PEI MAX (Polysciences Inc., Cat. #24765) in serum- and antibiotic-free DMEM and incubated for 10 min at RT. The DNA-PEI complexes were then added to cells in complete medium, and cells were maintained under standard culture conditions until further treatment.

### Flow cytometry

Flow cytometry analysis was performed using a BD FACS Canto II flow cytometer, and cell sorting was carried out using a BD FACS Aria II. Data were analyzed using FACSDiva software. For GFP-positive population analysis, dead cells and debris were excluded based on forward and side scatter profiles before fluorescence gating.

### Firefly/Renilla dual-luciferase assay

Cells were cotransfected with plasmids of interest, p5HRE-luc, and pRL-SV40 (Promega), cultured under normoxic or hypoxic conditions for 24 h, and lysed in Passive Lysis Buffer (Promega). Firefly and Renilla luciferase activities were measured using the Dual-Luciferase Assay Kit (Promega) according to the manufacturer’s instructions. Firefly luciferase activity was normalized to Renilla luciferase activity.

### RNA extraction and qRT-PCR

Total RNA was extracted using Sepasol-G (Nacalai Tesque, Cat. #09379) according to the manufacturer’s instructions. For conventional gene expression analysis, cDNA was synthesized using the PrimeScript RT Reagent Kit (Takara, Cat. #RR037A) with oligo(dT) and random hexamer primers. For the quantification of *ID1* and *DKK*1 lariat RNA, as well as total *ID1* and *DKK1* mRNA, gene-specific reverse primers identical to those used for qRT-PCR were used for cDNA synthesis at the final concentration of 0.1 μM. qRT-PCR was performed using TB Green Premix Ex Taq II (Takara, Cat. #RR820) on a TAKARA TP800 Thermal Cycler Dice Real Time System (Takara). β-actin or RPS18 was used as an internal control, and relative gene expression levels were calculated using the 2^-^ΔΔCt method. Primer sequences are listed in the Table S3. Lariat RNA levels were normalized to the corresponding total mRNA levels of the same gene or to RPS18, as indicated.

### Split luciferase complementation assay

HeLa cells (3 × 10^5^ cells/well in 6-well plates) were cotransfected with plasmids expressing fusion proteins of HIF-1α or DBR1 with either LgBiT or SmBiT. A total of 2 μg plasmid DNA was used per well at a 1:1 ratio. For negative controls, plasmids expressing fusion proteins with LgBiT were cotransfected with pcDNA4/EV-SmBiT-N. After 36 h, cells were reseeded into white 96-well plates at 8 × 10^3^ cells/well, with each tested condition seeded in triplicate wells, and incubated for an additional 8 h to allow attachment. Nano-Glo Live Cell Reagent (Promega, Cat. #N2012) was added to the cells according to the manufacturer’s instructions and incubated for 30 min at 37 °C. Luminescence was measured using a GloMax Navigator System (Promega, Cat. #GM2000).

### Western blotting

Cells were lysed in CelLytic M lysis buffer (Sigma-Aldrich, Cat. #C2978) supplemented with a protease inhibitor cocktail (Takara, Cat. #635673). Cytoplasmic and nuclear extracts were prepared using the NE-PER Nuclear and Cytoplasmic Extraction Reagents (Thermo Fisher Scientific, Cat. #78833), according to the manufacturer’s instructions. Equal amounts of protein were separated by SDS-PAGE and transferred to 0.45 μm polyvinylidene fluoride membranes (Cytiva, Cat. #15229824). Membranes were blocked with 5% skim milk in Tris-buffered saline with Tween-20 for 1 h and incubated overnight at 4 °C with primary antibodies against HIF-1α (1:200, BD Biosciences, Cat. #610959), HIF-2α (1:100, Santa Cruz Biotechnology, Cat. #sc-13596), DBR1 (1:50,000, Proteintech, Cat. #66951-1-Ig), HIF-1β (1:500, Novus Biologicals, Cat. #NB100-124), and β-actin (1:250, Santa Cruz Biotechnology, Cat. #sc-47778). After incubation with HRP-conjugated secondary antibodies (ECL anti-mouse IgG, Cytiva, Cat. #NA931V), signals were detected using ECL Prime Western Blotting Detection Reagents (Cytiva, Cat. #RPN2232). Chemiluminescence was detected using an Amersham Imager 600. Antibody specificity was validated based on the manufacturer’s information and by detection of proteins at their expected molecular weights.

### ChIP

Cells (1 × 10^6^ cells/100 mm dish) were transfected with the indicated plasmids and cultured under normoxic or hypoxic conditions for 24 h. Cells were crosslinked with 1% formaldehyde (Sigma-Aldrich, Cat. #F8775) for 2 min at RT, and the reaction was quenched with 125 mM glycine (Fujifilm, Cat. #077-00735). Chromatin was sheared by sonication (16 cycles of 30 s ON and 30 s OFF at high power, Bioruptor UCW-210), and the resulting sheared chromatin were incubated overnight at 4 °C with an anti-HIF-1α antibody (3 μg/sample, Abcam, Cat. #H1alpha67). A negative control was prepared using the same amount of purified mouse IgG1 κ isotype control (BD Biosciences, Cat. #554121). Protein-DNA complexes were captured using Dynabeads Protein G (Thermo Fisher Scientific, Cat. #10007D), followed by washing and elution. Crosslinks were reversed by incubation at 65 °C overnight. Samples were then treated with RNase A (Thermo Fisher Scientific, Cat. #EN0531) and proteinase K (Thermo Fisher Scientific, Cat. #26160), and DNA was purified using a Qiagen PCR Purification Kit according to the manufacturer’s instructions. Purified DNA was eluted in TE buffer. Enrichment of target regions was quantified by qPCR and normalized to input DNA. Primer sequences are listed in the Table S3.

### Chemotherapy response assay and colorimetric cell viability assay

Cells (1 × 10^3^ cells/well in 96-well plate) were allowed to attach overnight. Cells were then cultured under hypoxic or normoxic conditions for 24 h, followed by treatment with drug-containing medium for an additional 24 h under the corresponding oxygen condition. Camptothecin (Fujifilm, Cat. #038-18191) and carboplatin (Fujifilm, Cat. #033-25231) were diluted to the final concentration of 0.8 μM and 100 μM in DMEM, respectively. The cell viability was determined using Cell Count Reagent SF (Nacalai Tesque, Cat. #07553-44) according to the manufacturer’s instructions. Viability was normalized to untreated cells in the same oxygen conditions.

### Statistical analysis

Statistical analyses were performed using GraphPad Prism 9.0 (GraphPad Software). Quantitative data are presented as the mean ± SD of three technical replicates from a representative experiment. Each experiment was independently repeated at least three times with similar results to confirm reproducibility. Comparisons between two groups were performed using a two-tailed unpaired Student’s *t* test. This test was selected because the analyses were designed to compare the means of two independent experimental groups, and a two-tailed test was used because differences in either direction were considered biologically possible. A *p* value of < 0.05 was considered statistically significant. Statistical significance is indicated as follows: ns, not significant; ∗*p* < 0.05; ∗∗*p* < 0.01; and ∗∗∗*p* < 0.001.

## Data availability

The data supporting the findings of this study are available within the article and its Supporting Information. Additional data are available from the corresponding author upon reasonable request.

## Supporting information

This article contains supporting information.

## Conflict of interest

The authors declare that they have no conflicts of interest with the contents of this article.
