# Supplementary material for: The lariat RNA debranching enzyme DBR1 enhances HIF-1 signaling and chemoresistance at the expense of lariat RNA processing
Source: J Biol Chem. 2026 Aug 25;302(10):113484. doi: 10.1016/j.jbc.2026.113484 (PMC13629192; doi:10.1016/j.jbc.2026.113484)
Supplement: Supplementary file 1 [file mmc1.pdf]

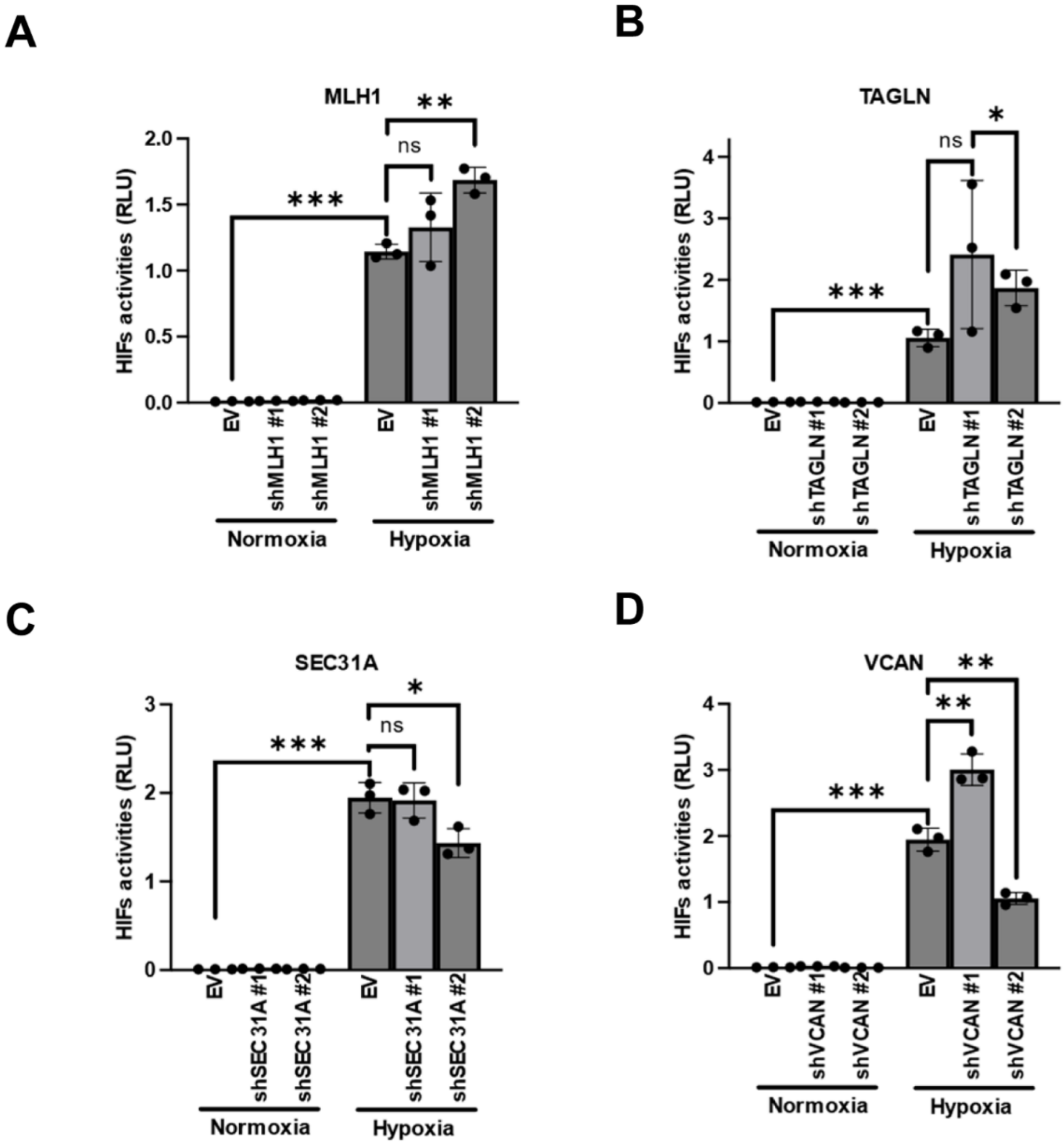

Supplementary Figure S1.

Validation of candidate HIF regulators identified by shRNA screening.

(A-D) HeLa cells were transduced with two independent shRNAs targeting MLH1 (A), TAGLN (B), SEC31A (C), or VCAN (D), using the sequences listed in Supplementary Table S2. Candidate gene-knockdown cells (1×10^4 cells/well in a 24-well plate) were co-transfected with the p5HRE-luc reporter and a Renilla luciferase control plasmid, followed by exposure to normoxic or hypoxic conditions for 24 h. HIF-dependent transcriptional activity was assessed by dual-luciferase assay, with firefly luciferase activity normalized to Renilla luciferase activity. HeLa cells transduced with the control pRSI9-U6-(sh)-UbiC-TagRFP-2A-Puro vector were used as the empty-vector control (EV).

Data represent the mean ± SD (n = 3). Statistical significance was determined using a two-tailed Student's t-test. ns, not significant; *$p < 0.05$, **$p < 0.01$, ***$p < 0.001$.

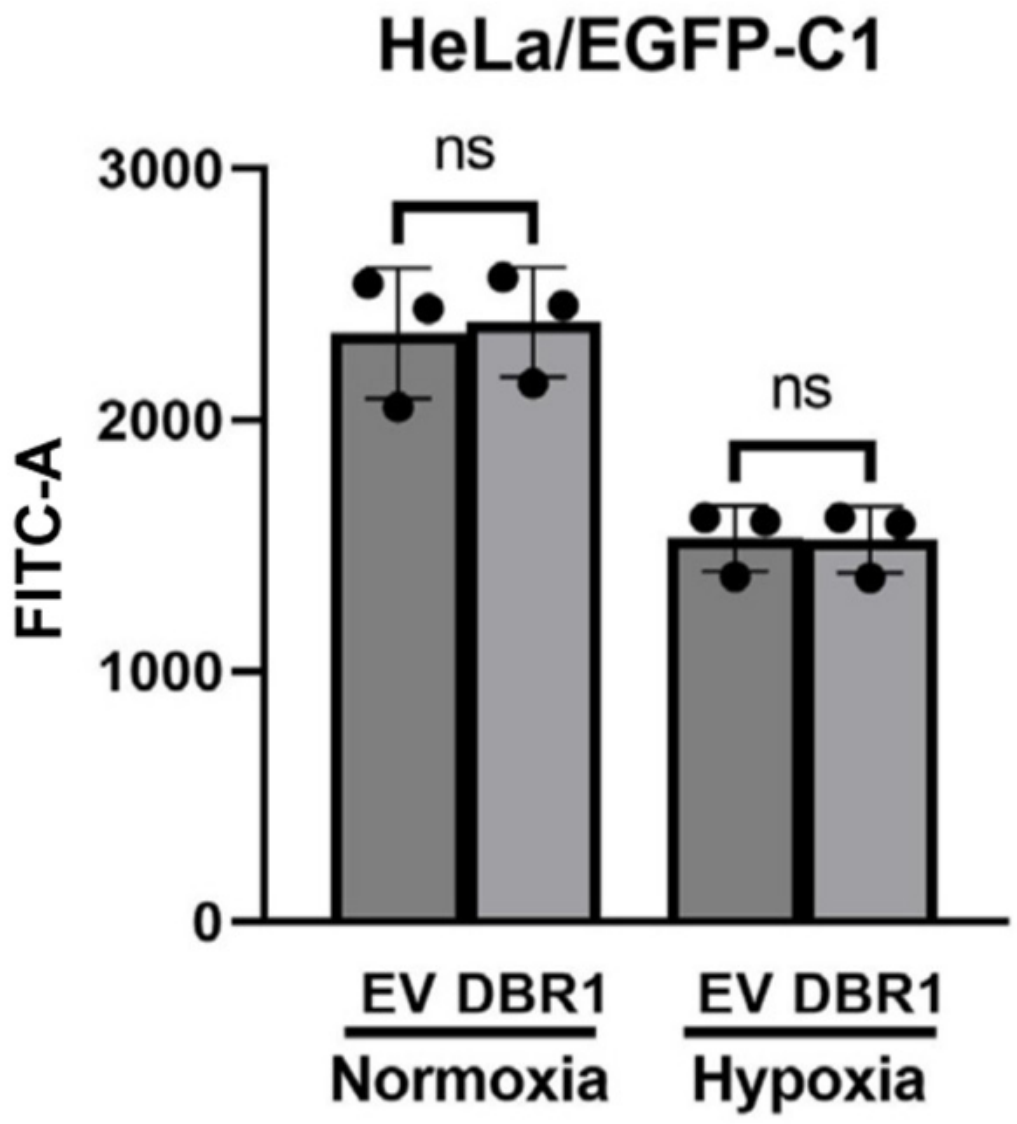

Supplementary Figure S2.

DBR1 overexpression does not affect basal EGFP fluorescence.

HeLa/EGFP-C1 cells stably expressing EGFP (1 × 10^5 cells/3.5-cm dish) were transfected with either pCDH-DBR1-puro or pCDH/EF1-MCS-IRES-Puro as a negative control (EV) and cultured under normoxic or hypoxic conditions for 24 h. EGFP fluorescence (FITC-A) was measured by flow cytometry using a FACSCanto II.

Data represent the mean ± SD (n = 3). Statistical significance was determined using a two-tailed Student's t-test. ns, not significant.

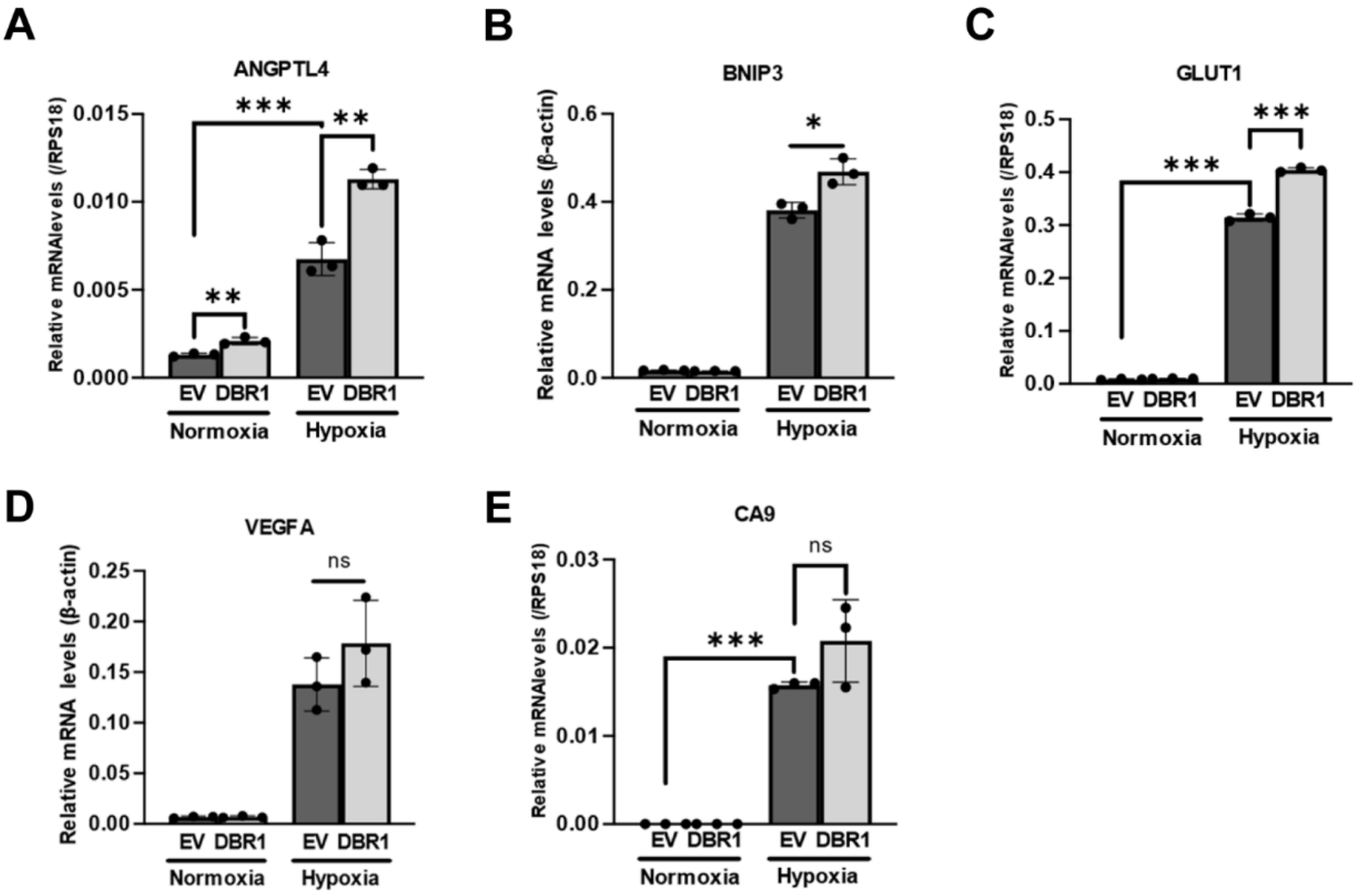

Supplementary Figure S3.

DBR1 differentially enhances the hypoxic induction of HIF-1 target genes in HeLa cells.

Total RNA was prepared from the same cells as those used in Figure 1E. The mRNA expression levels of ANGPTL4 (A), BNIP3 (B), GLUT1 (C), VEGFA (D), and CA9 (E) were quantified by RT-qPCR and normalized to β-actin or RPS18 expression. DBR1 overexpression was confirmed by Western blotting as shown in Figure 1E.

Data represent the mean ± SD (n = 3). Statistical significance was determined using a two-tailed Student's t-test. ns, not significant; $*p < 0.05$, $**p < 0.01$, $***p < 0.001$.

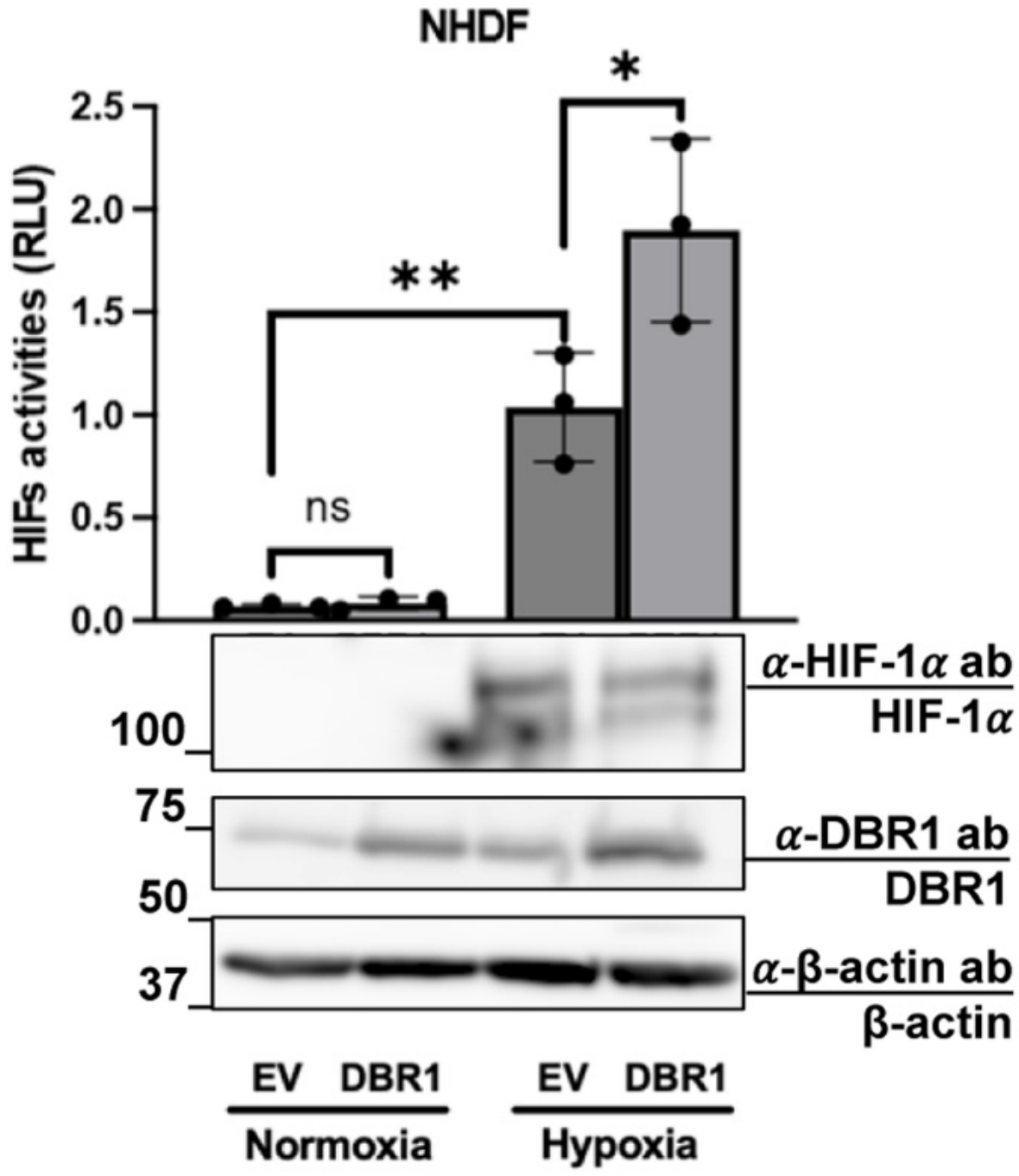

Supplementary Figure S4.

DBR1 overexpression enhances HIF-dependent transcriptional activity in NHDF cells.

NHDF cells (2 × 10^4 cells/well in a 24-well plate) were co-transfected with a DBR1 expression plasmid or its empty vector (EV), together with p5HRE-luc and a Renilla luciferase control plasmid. Following exposure to hypoxic conditions for 15 h, luciferase activity was measured, and firefly luciferase activity was normalized to Renilla luciferase activity. DBR1 overexpression was confirmed by Western blotting.

Data represent the mean ± SD (n = 3). Statistical significance was determined using a two-tailed Student's t-test. **p < 0.001.

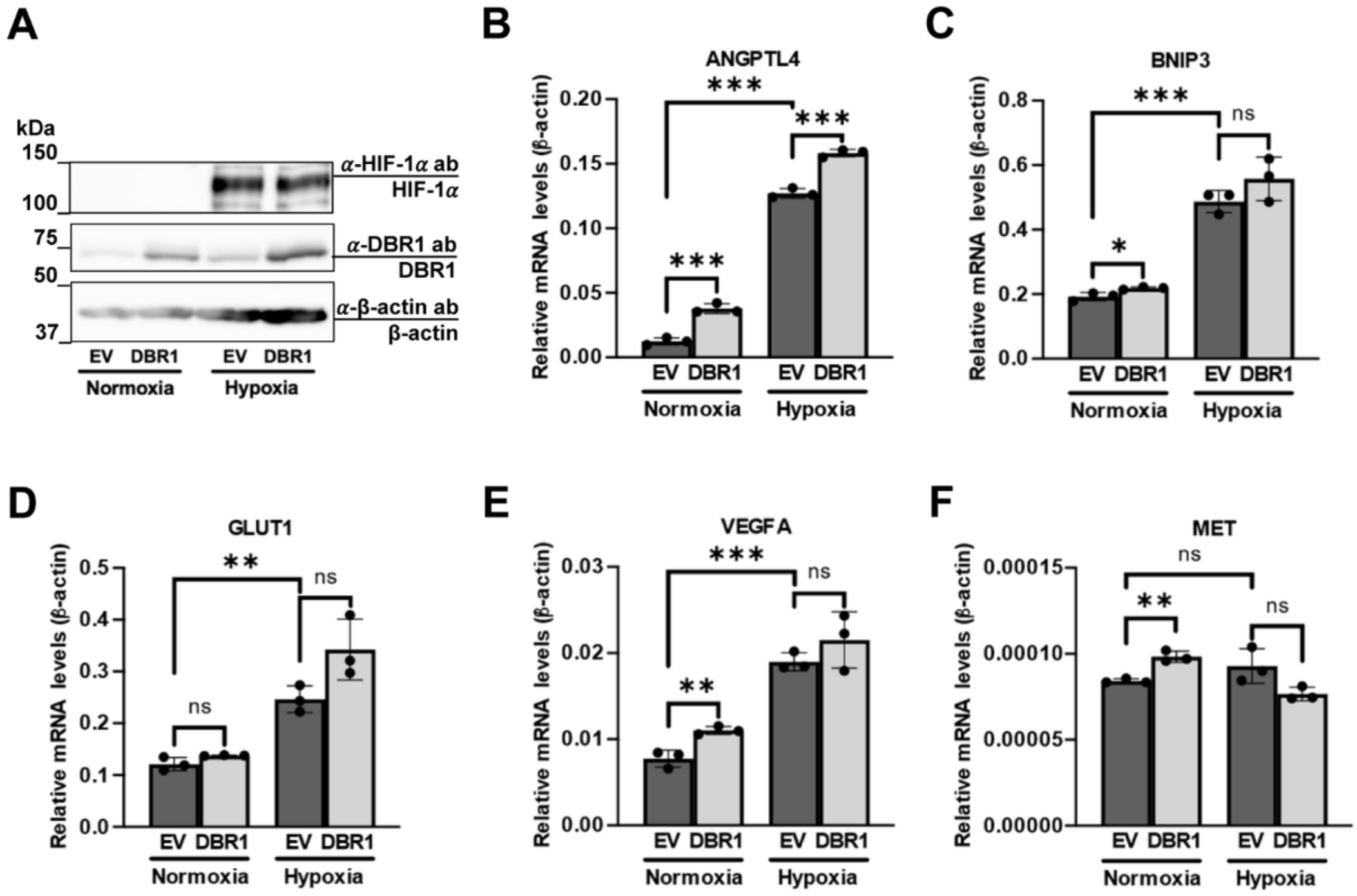

Supplementary Figure S5.

Effects of DBR1 overexpression on HIF-1 target gene expression in NHDF cells.

NHDF cells (5 × 10^5 cells/well in a 6-well plate) were co-transfected with a DBR1 expression plasmid or its empty vector (EV) and cultured under normoxic or hypoxic conditions for 18 h before total RNA extraction. (A) Cell lysates were analyzed by Western blotting using the indicated antibodies. The mRNA expression levels of ANGPTL4 (B), BNIP3 (C), GLUT1 (D), VEGFA (E), and MET (F) were quantified by RT-qPCR and normalized to β-actin expression.

Data represent the mean ± SD (n = 3). Statistical significance was determined using a two-tailed Student's t-test. ns, not significant; *$p < 0.05$, **$p < 0.01$, ***$p < 0.001$.

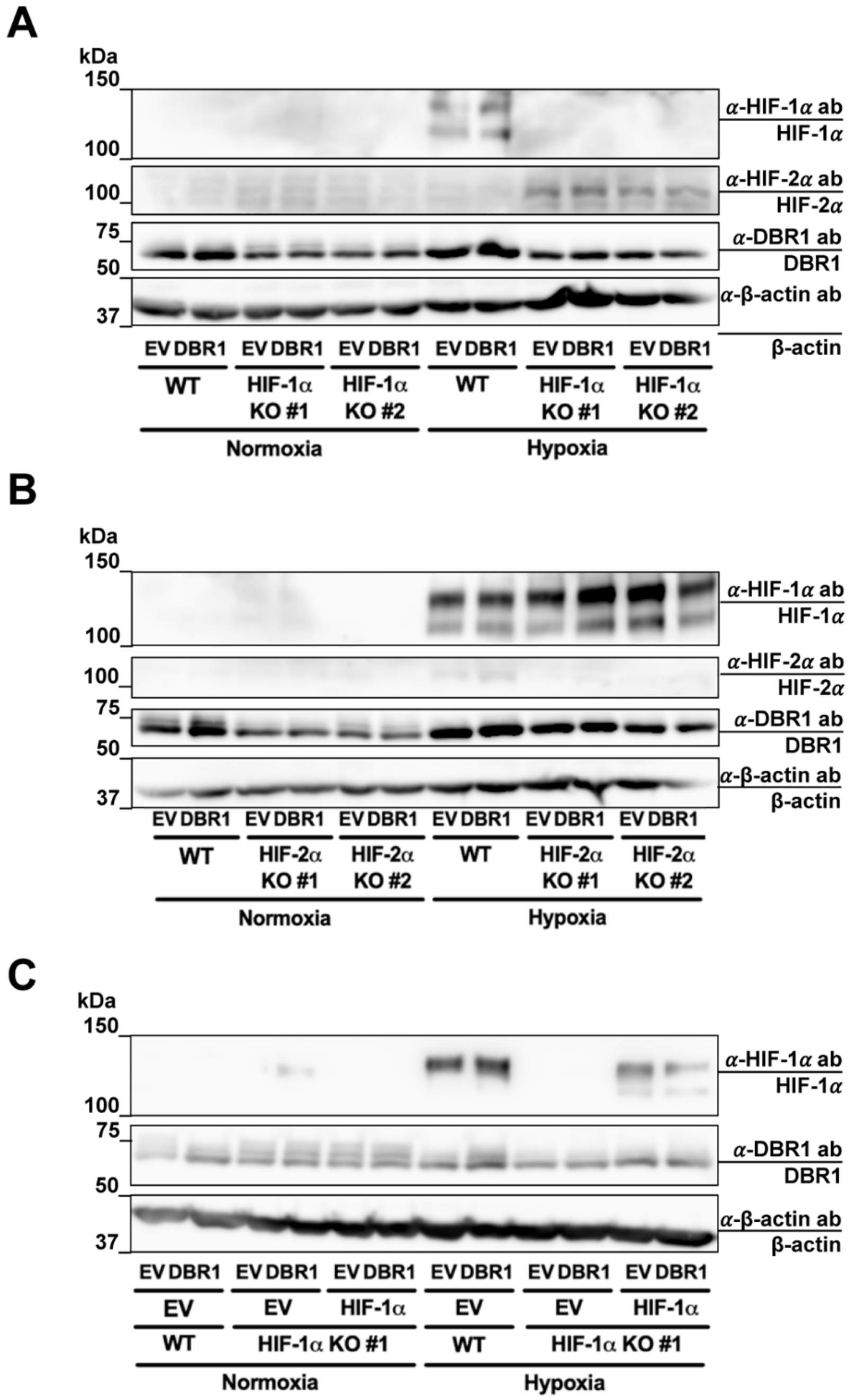

Supplementary Figure S6.

Validation of HIF-1α and HIF-2α knockout, HIF-1α reconstitution, and DBR1 overexpression in HeLa cells.

Cell lysates harvested from the same cells used in Figure 2A (A), Figure 2B (B), and Figure 2C (C) were analyzed by Western blotting using the indicated antibodies.

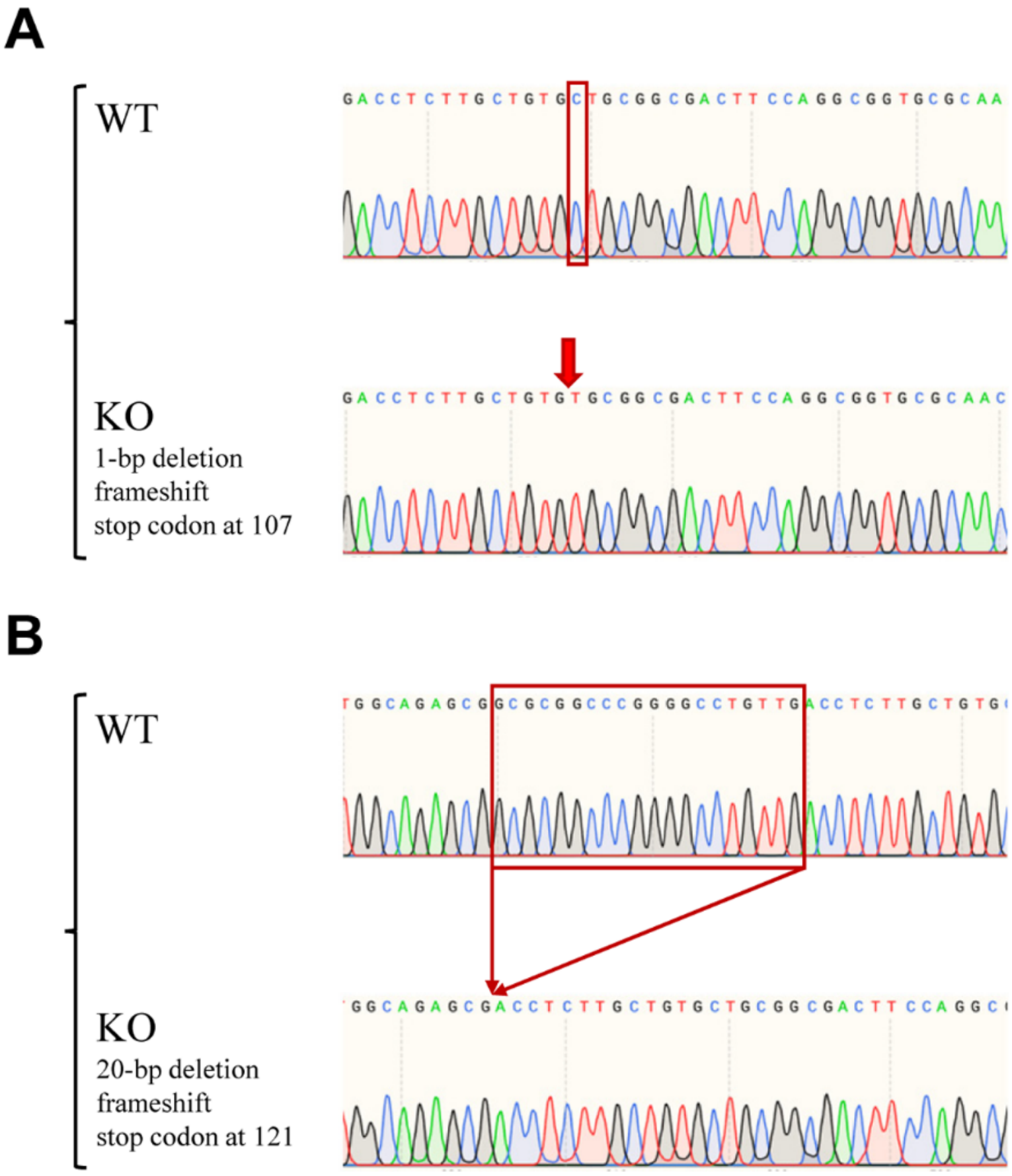

Supplementary Figure S7.

Genotypic validation of the DBR1-KO HeLa clone.

(A, B) Sanger sequencing analysis of the two DBR1 alleles in the DBR1-KO HeLa clone. A 1-bp deletion in one allele (A) and a 20-bp deletion in the other allele (B) resulted in frameshift mutations and premature stop codons at amino acid positions 107 and 121, respectively.

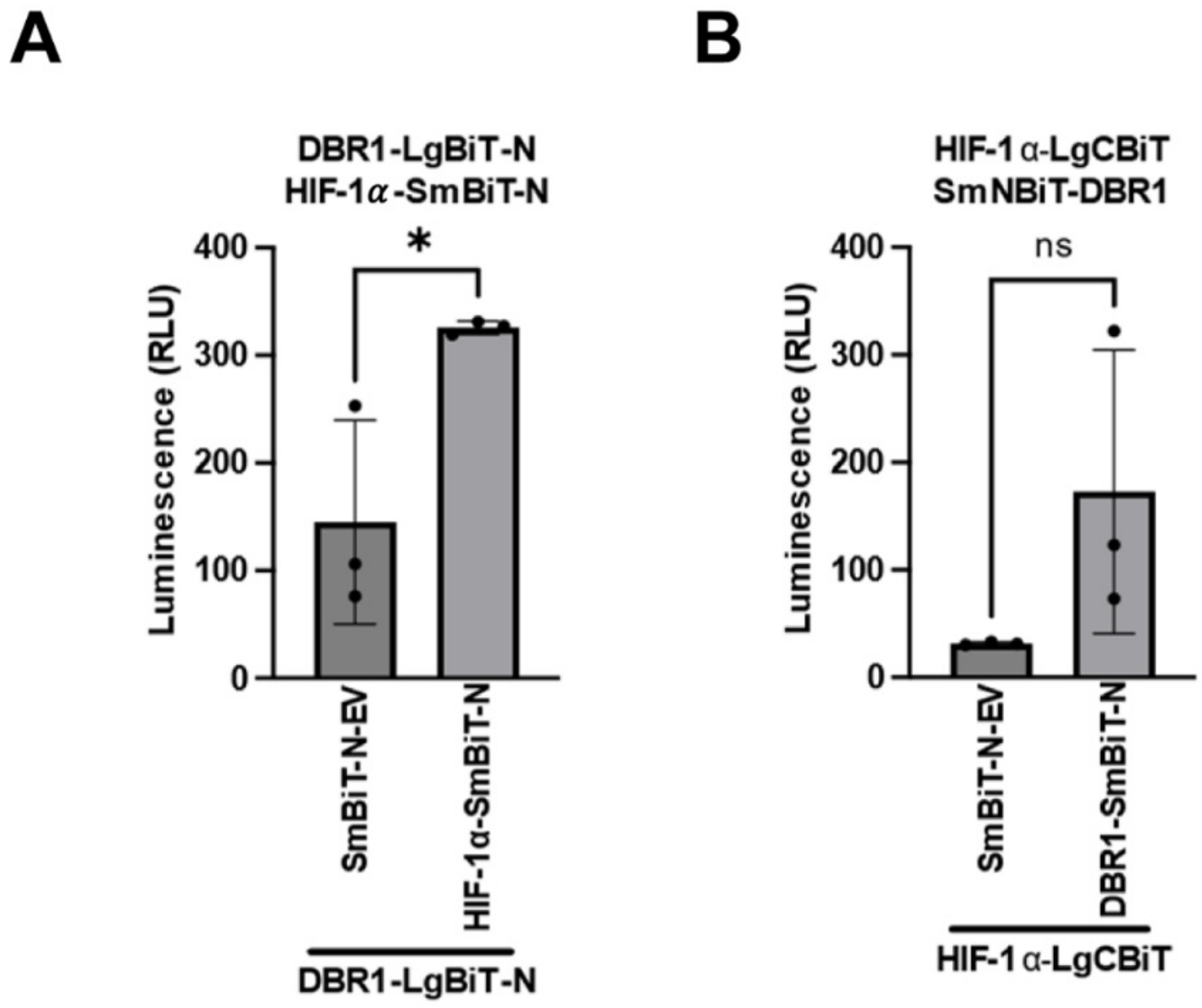

Supplementary Figure S8.

DBR1 interacts with HIF-1α in primary NHDF cells.

(A, B) NHDF cells (5 × 10^4 cells/well in a 12-well plate) were co-transfected with the DBR1-LgBiT-N expression plasmid and either the HIF-1α-2A-SmBiT-N expression plasmid or SmBiT-N as a negative control (A), or with the HIF-1α-2A-LgBiT-N expression plasmid and either the DBR1-SmBiT-N expression plasmid or SmBiT-N as a negative control (B). Twenty-four hours after transfection, cells were reseeded (8 × 10^3 cells/well in a 96-well plate) for split luciferase complementation analysis. Luminescence was measured to assess the interaction between DBR1 and HIF-1α.

Data represent the mean ± SD (n = 3). Statistical significance was determined using a two-tailed Student's t-test. **$p < 0.001$.

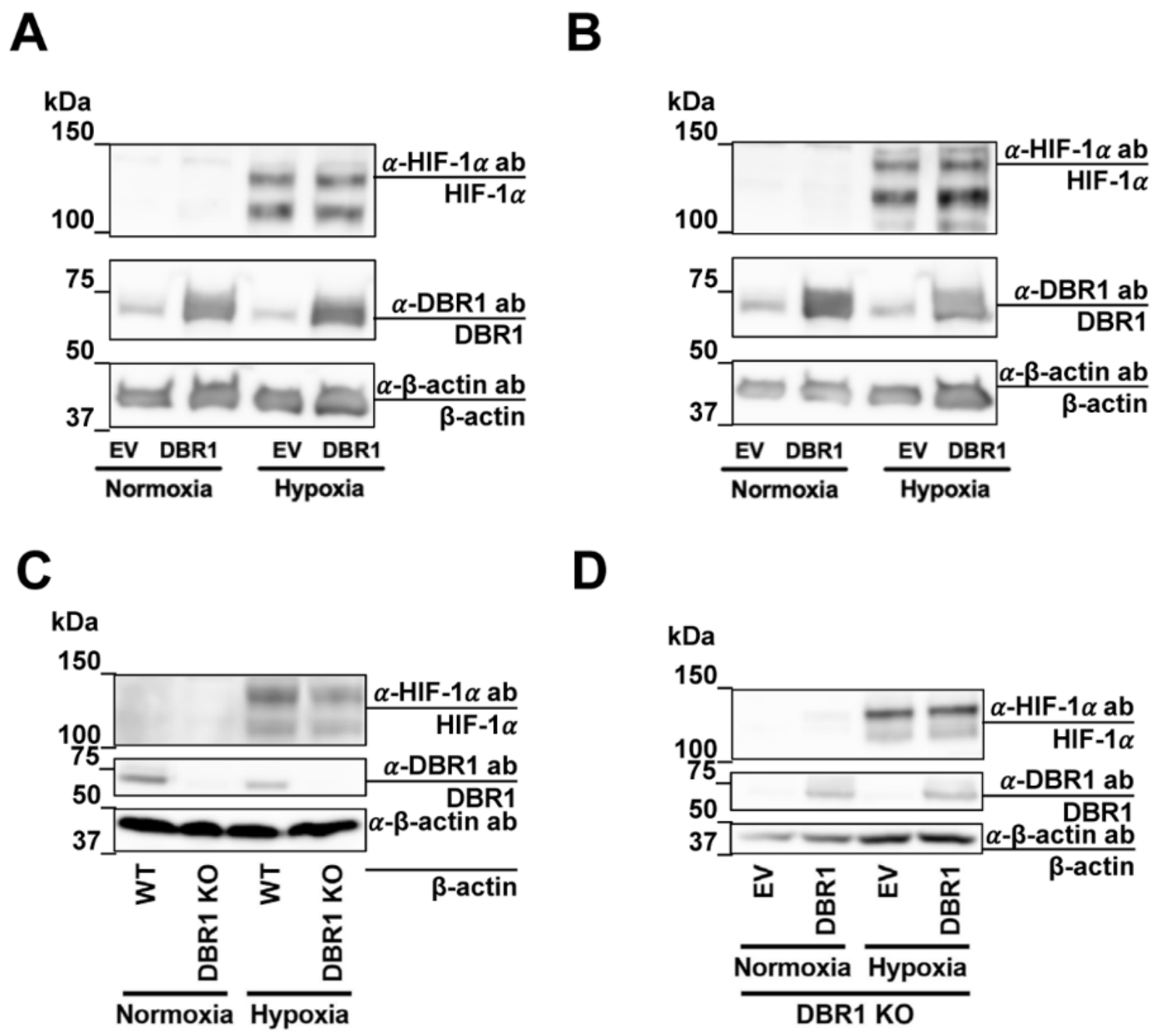

Supplementary Figure S9.

Validation of DBR1 overexpression, knockout, and reconstitution in cells used for the experiments shown in Figure 4E–H.

Cell lysates harvested from the same cells used in Figure 4E (A), Figure 4F (B), Figure 4G (C), and Figure 4H (D) were analyzed by Western blotting using the indicated antibodies.

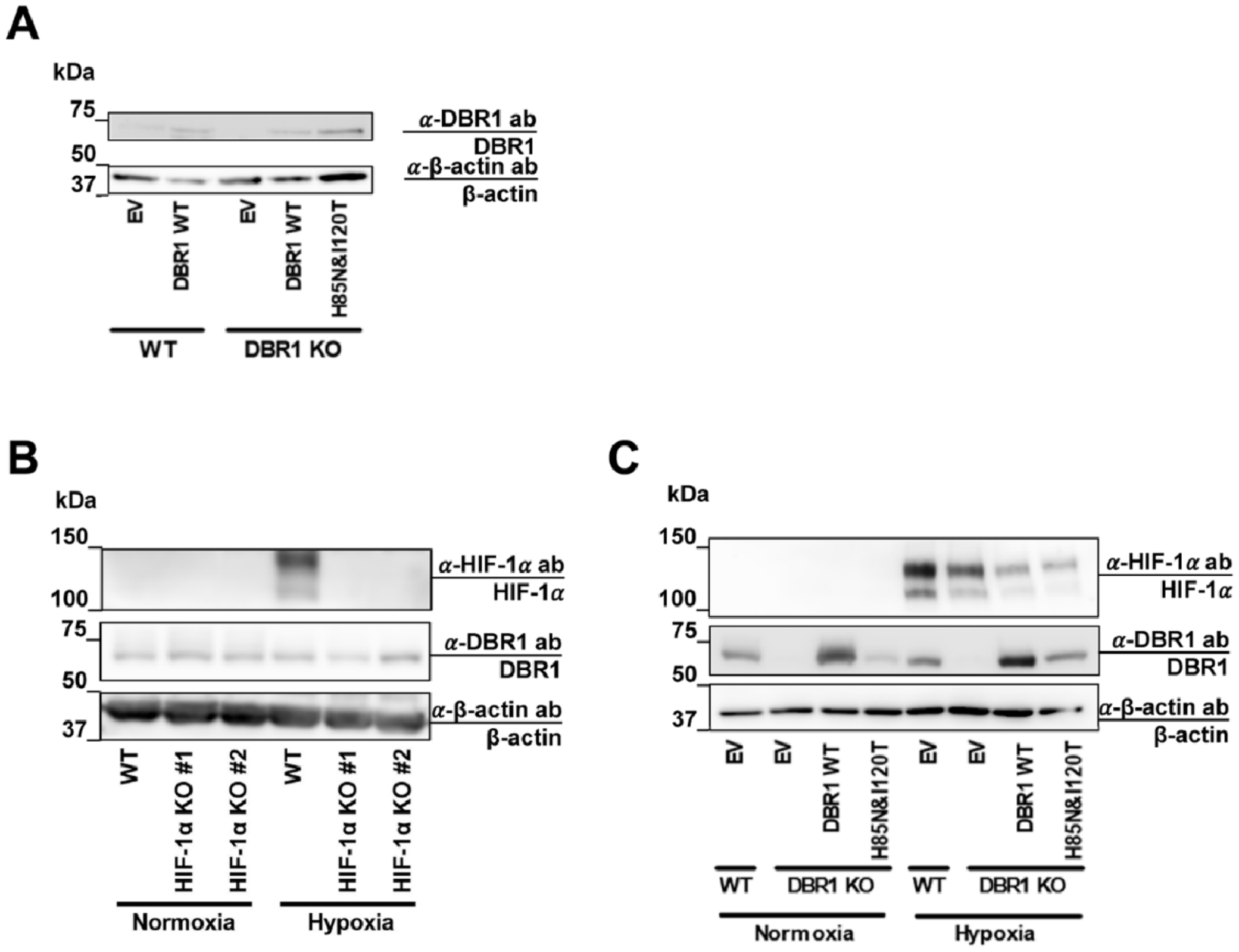

Supplementary Figure S10.

Validation of DBR1 and HIF-1α expression in cells used for the experiments shown in Figures 5 and 6.

Cell lysates harvested from the same cells used in Figures 5B and 5C (A), Figures 6A and 6B (B), and Figures 6E and 6F (C) were analyzed by Western blotting using the indicated antibodies.

Fig. 1B

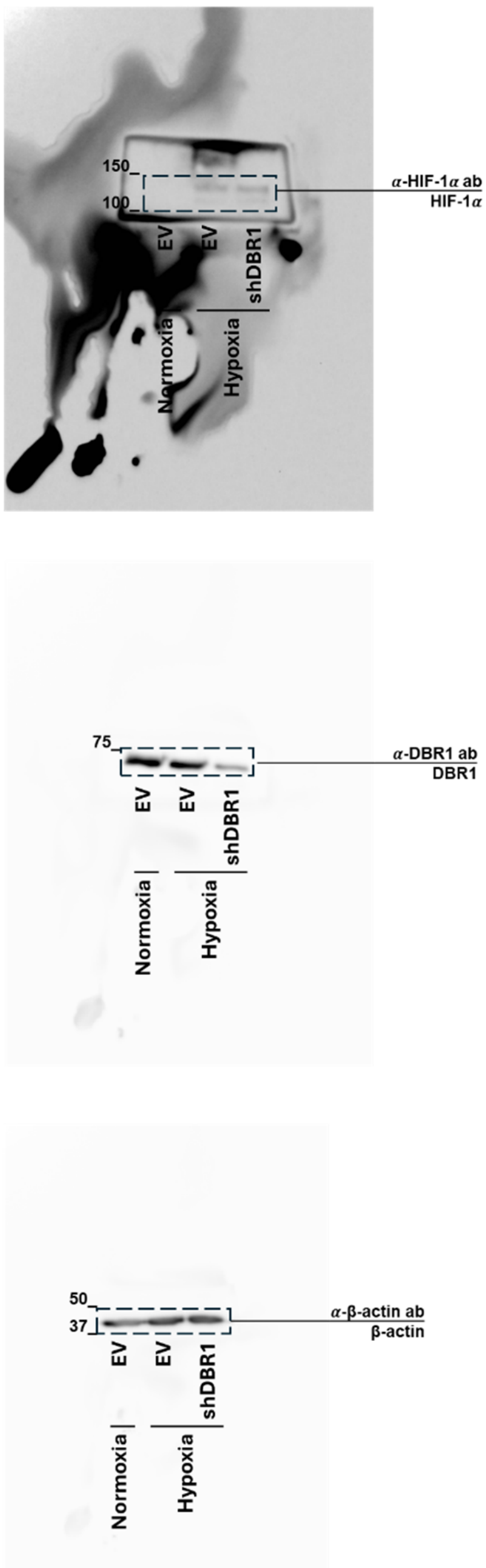

Fig. 1C

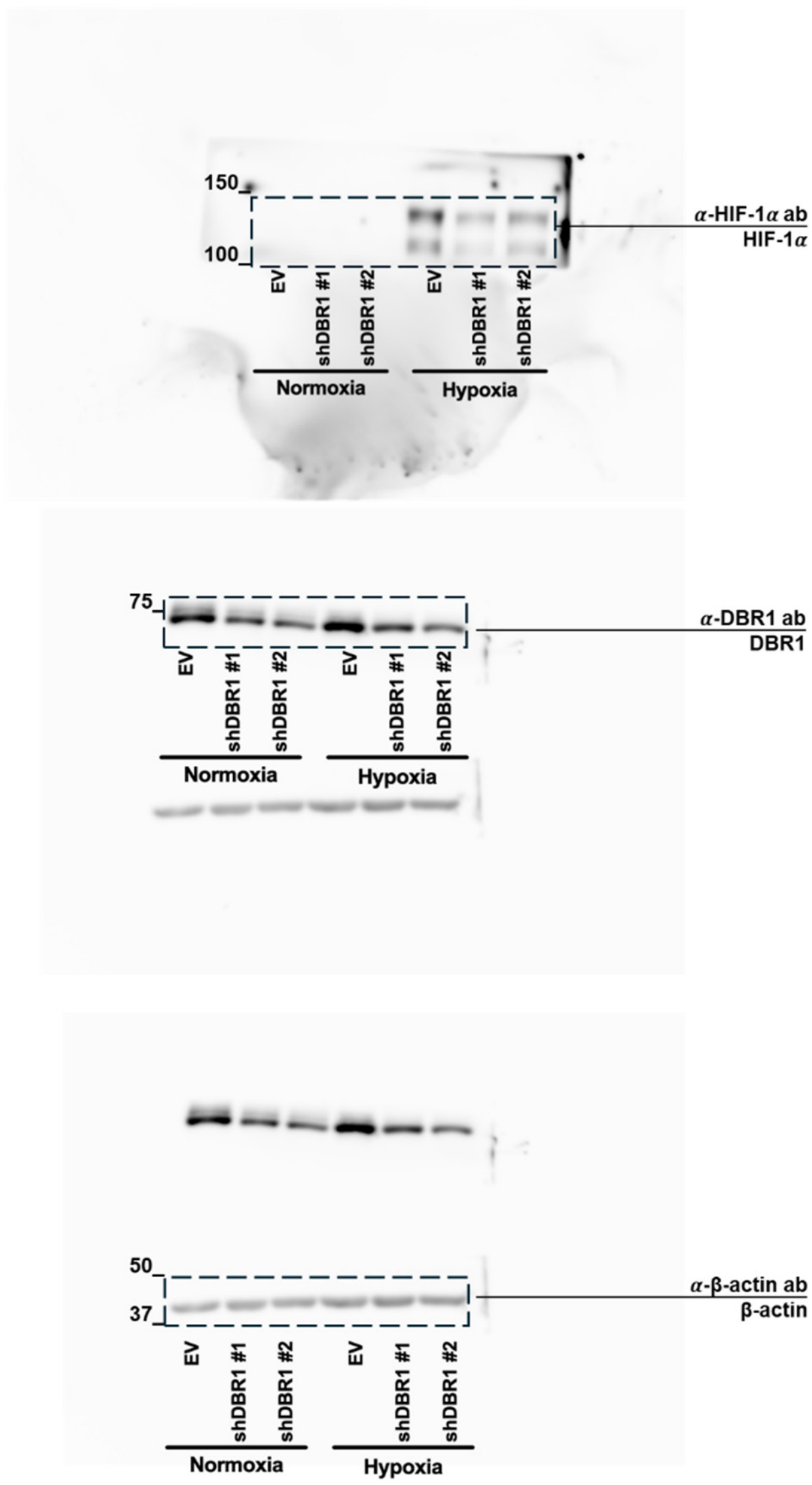

Supplementary Figure S11-1. Uncropped Western Blot Images

Fig. 1D

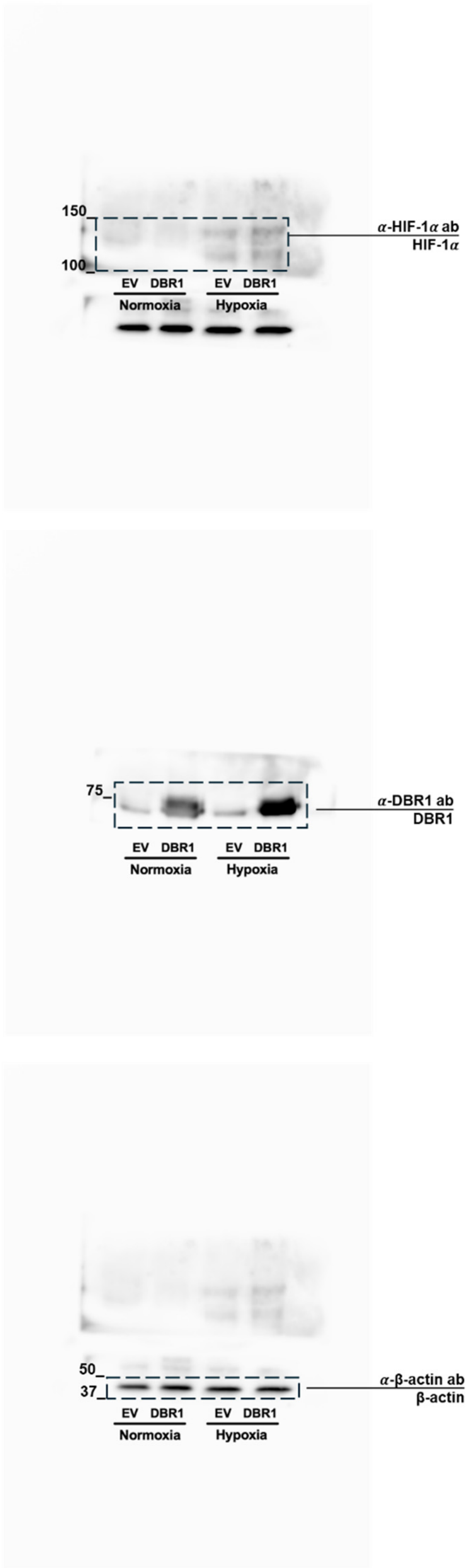

Fig. 1E

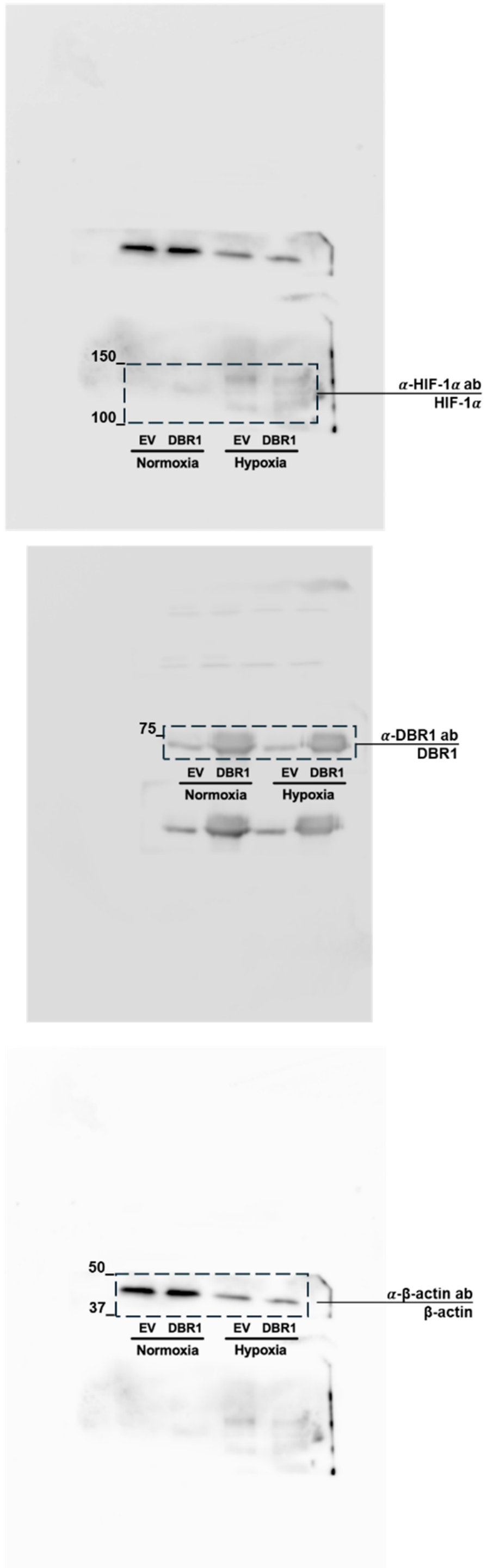

Supplementary Figure S11-2. Uncropped Western Blot Images

Fig. 1F

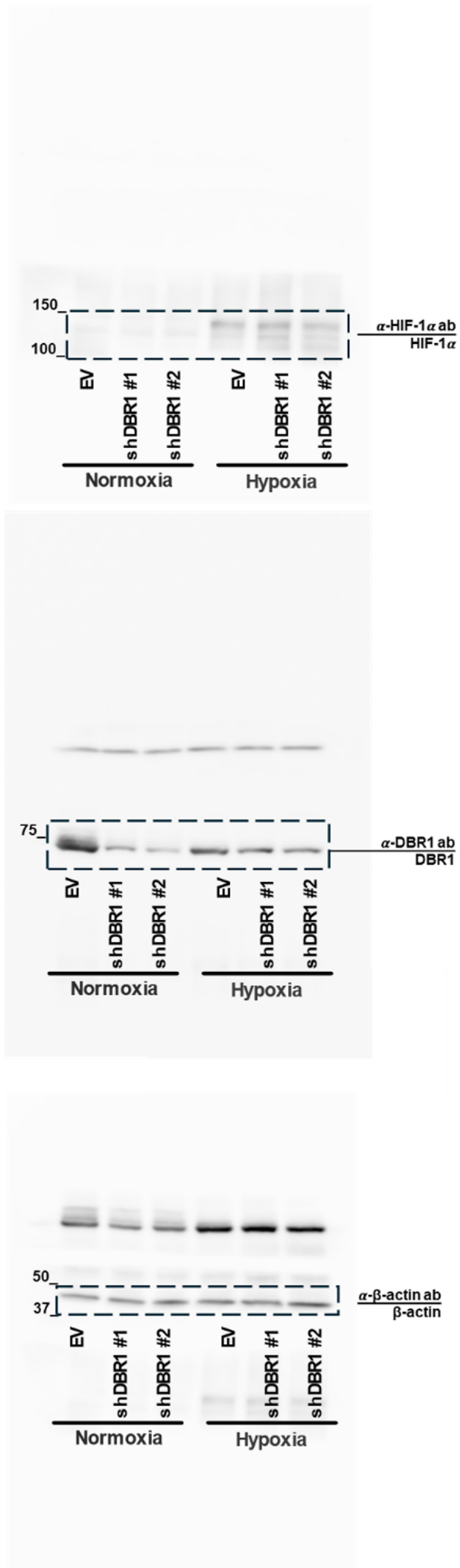

Fig. 3C

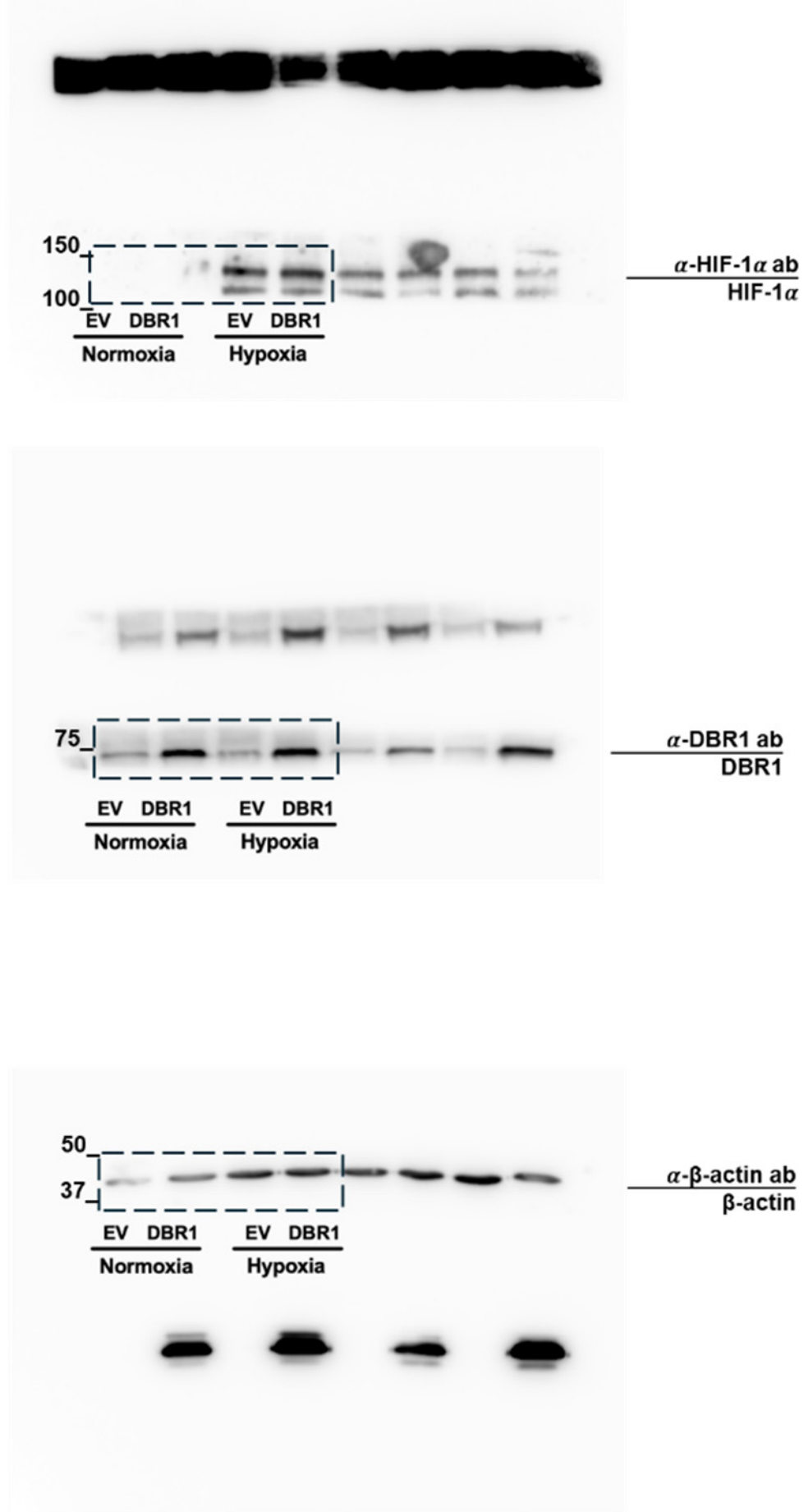

Supplementary Figure S11-3. Uncropped Western Blot Images

Fig. 3D

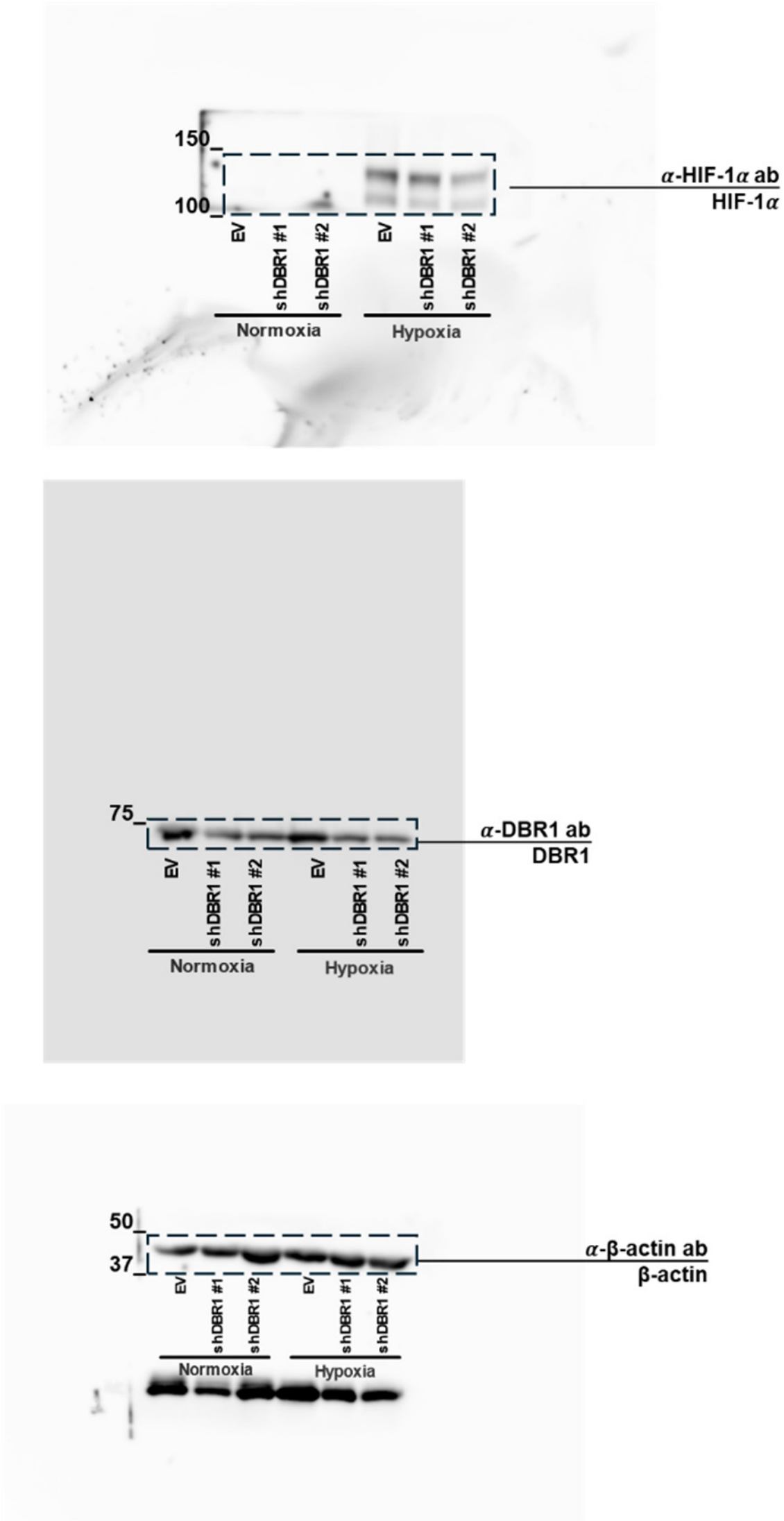

Fig. 3E

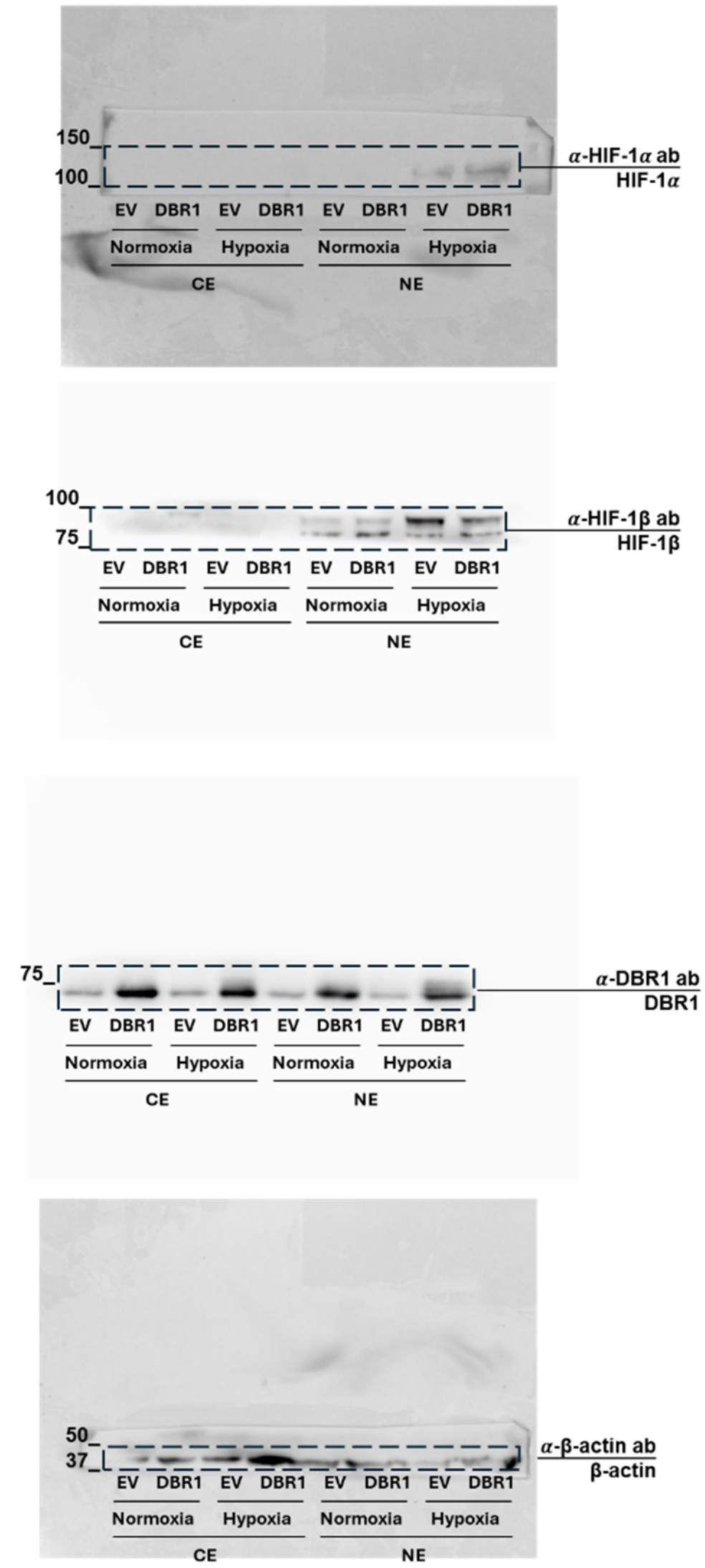

Supplementary Figure S11-4. Uncropped Western Blot Images

Fig. 3F

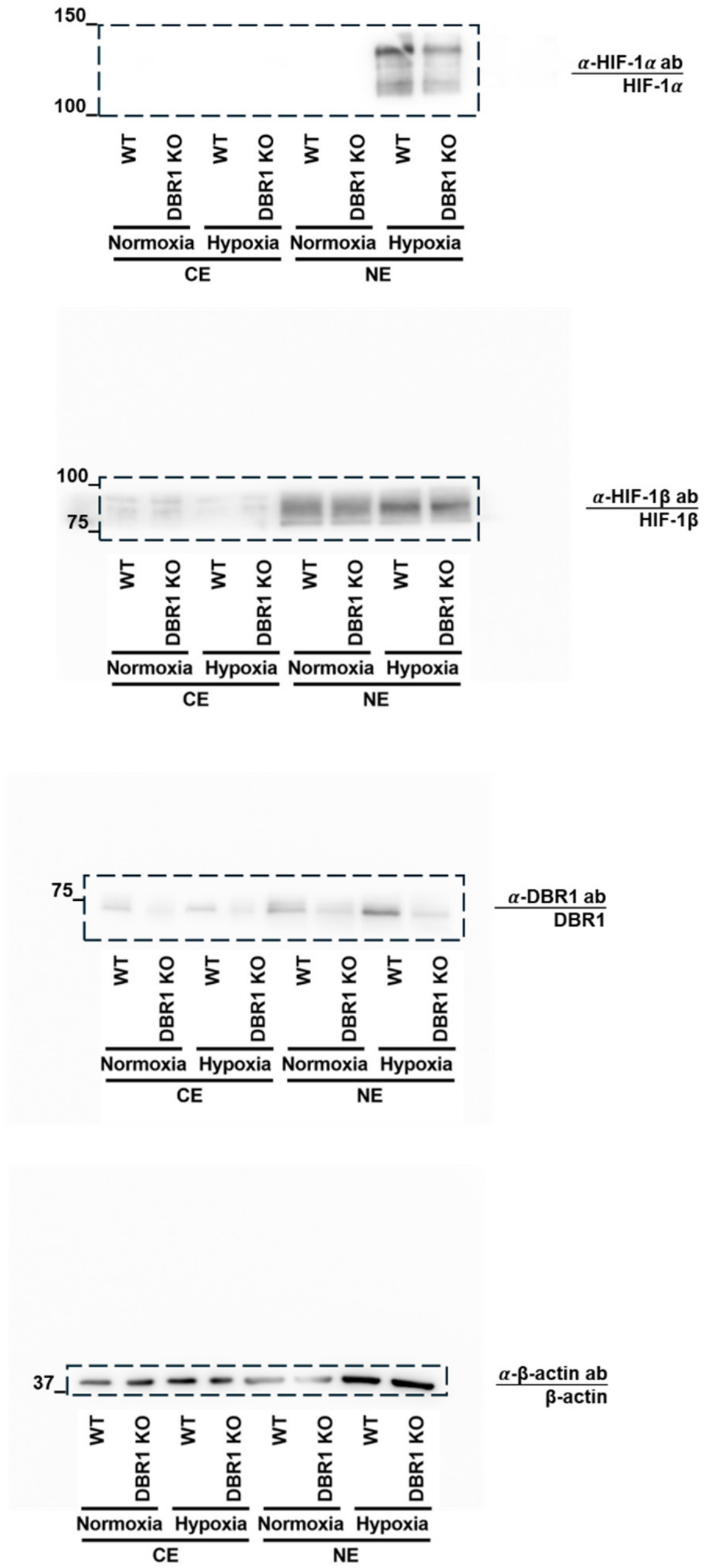

Fig. 4B

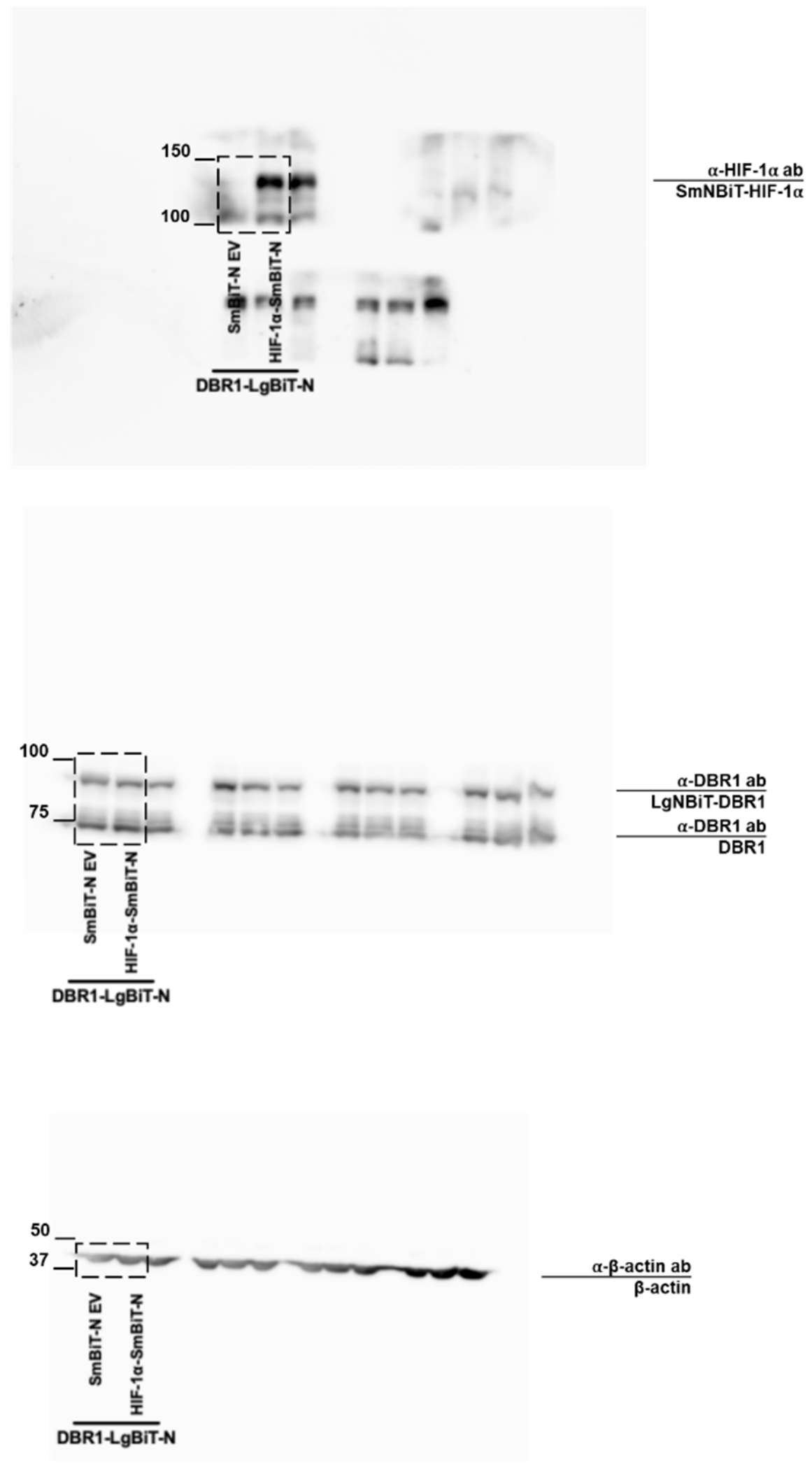

Supplementary Figure S11-5. Uncropped Western Blot Images

Fig. 4D

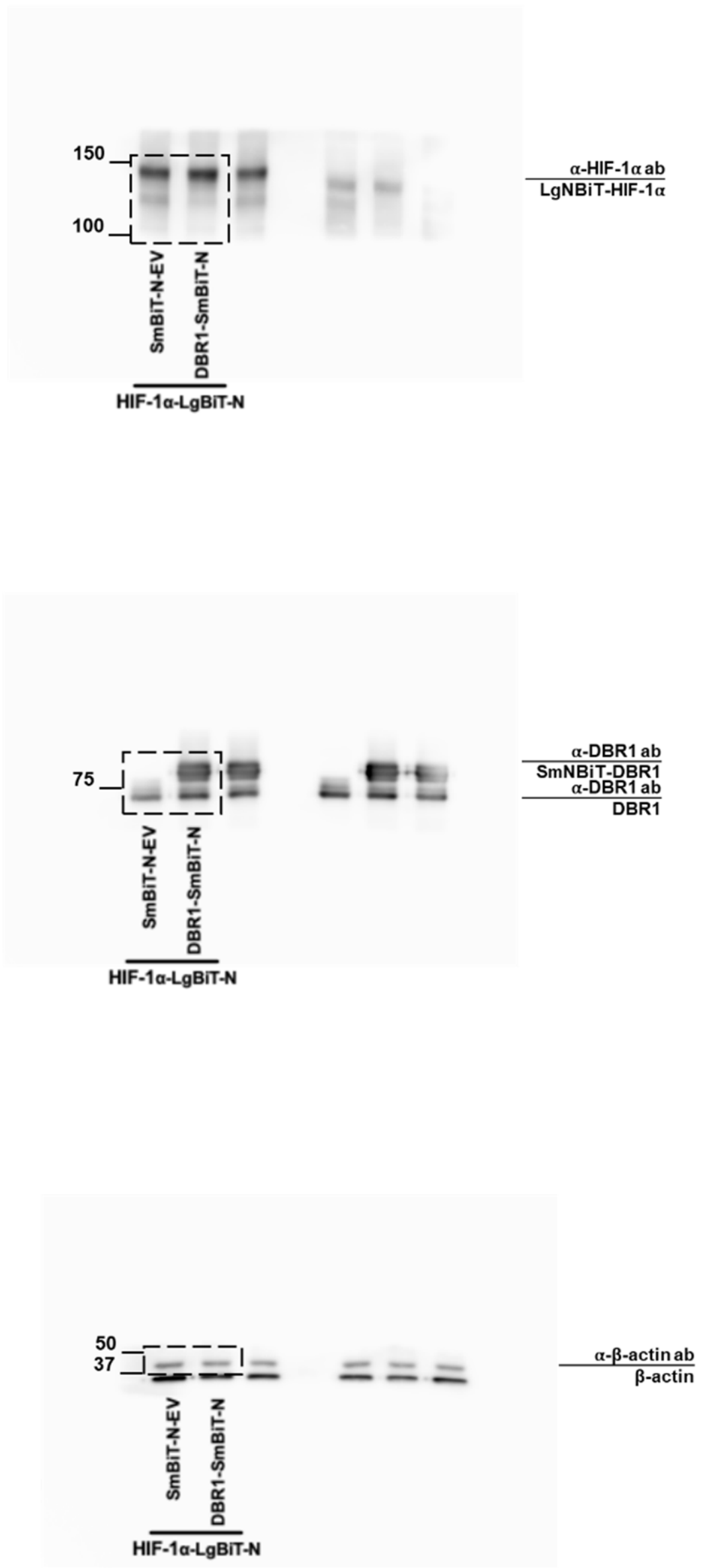

Fig. 5D

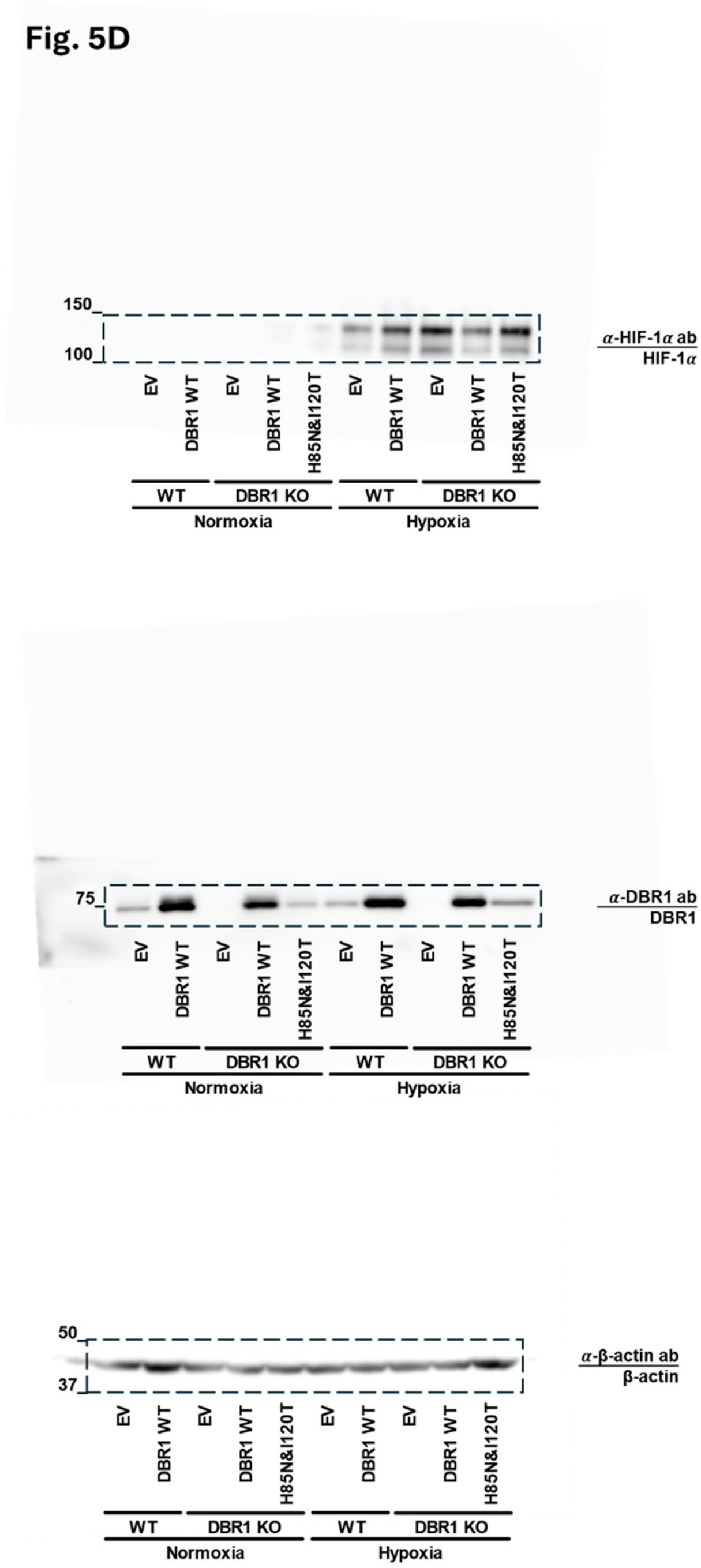

Supplementary Figure S11-6. Uncropped Western Blot Images

Sup. Fig. 4

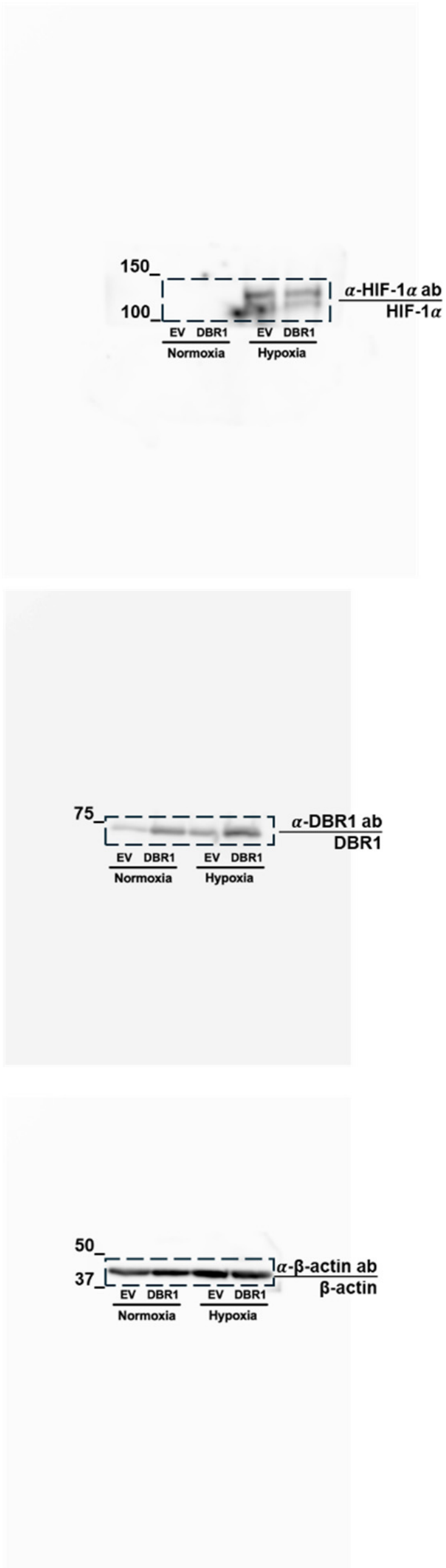

Sup. Fig. 5A

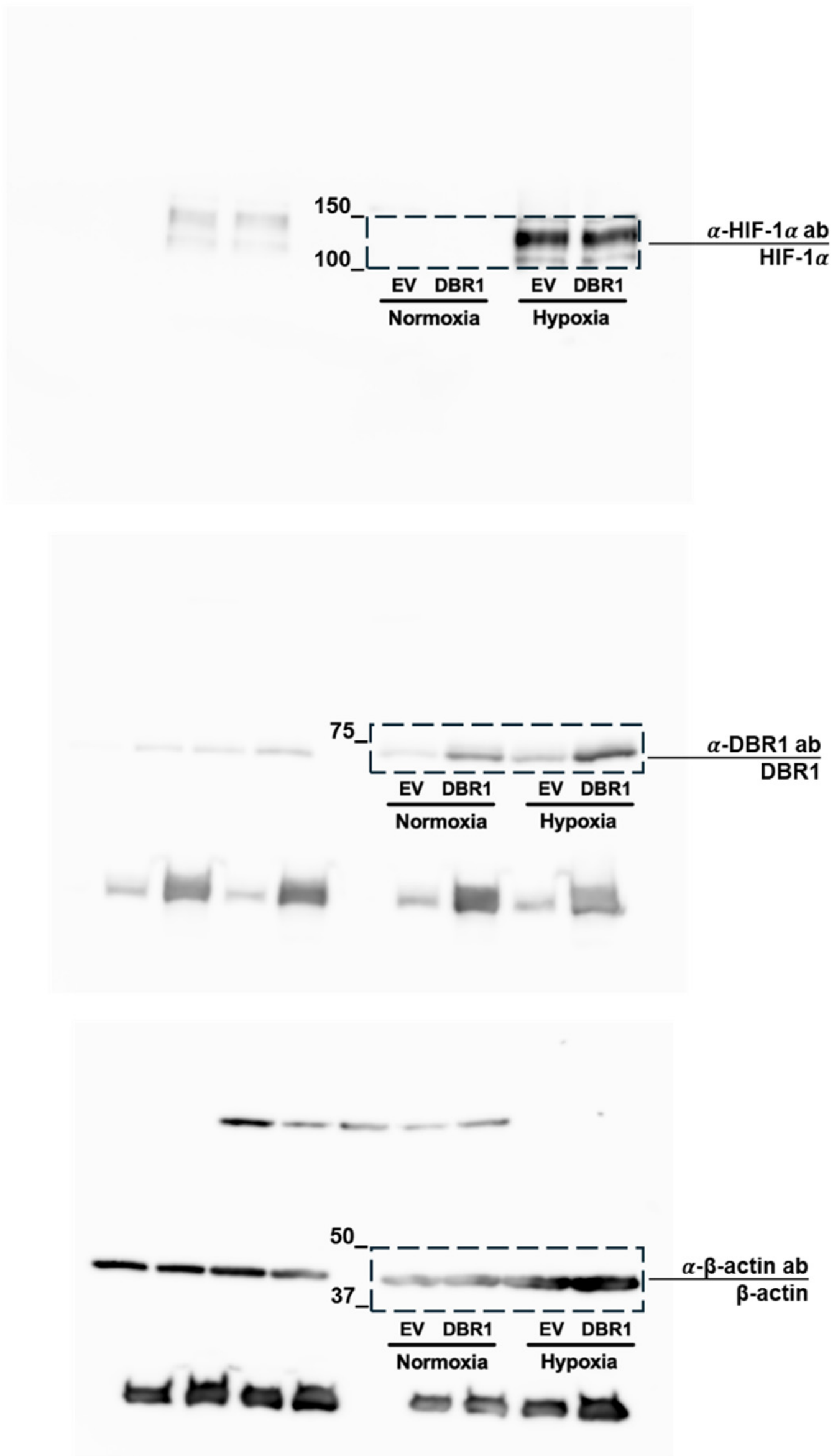

Supplementary Figure S11-7. Uncropped Western Blot Images

Sup. Fig. 6A

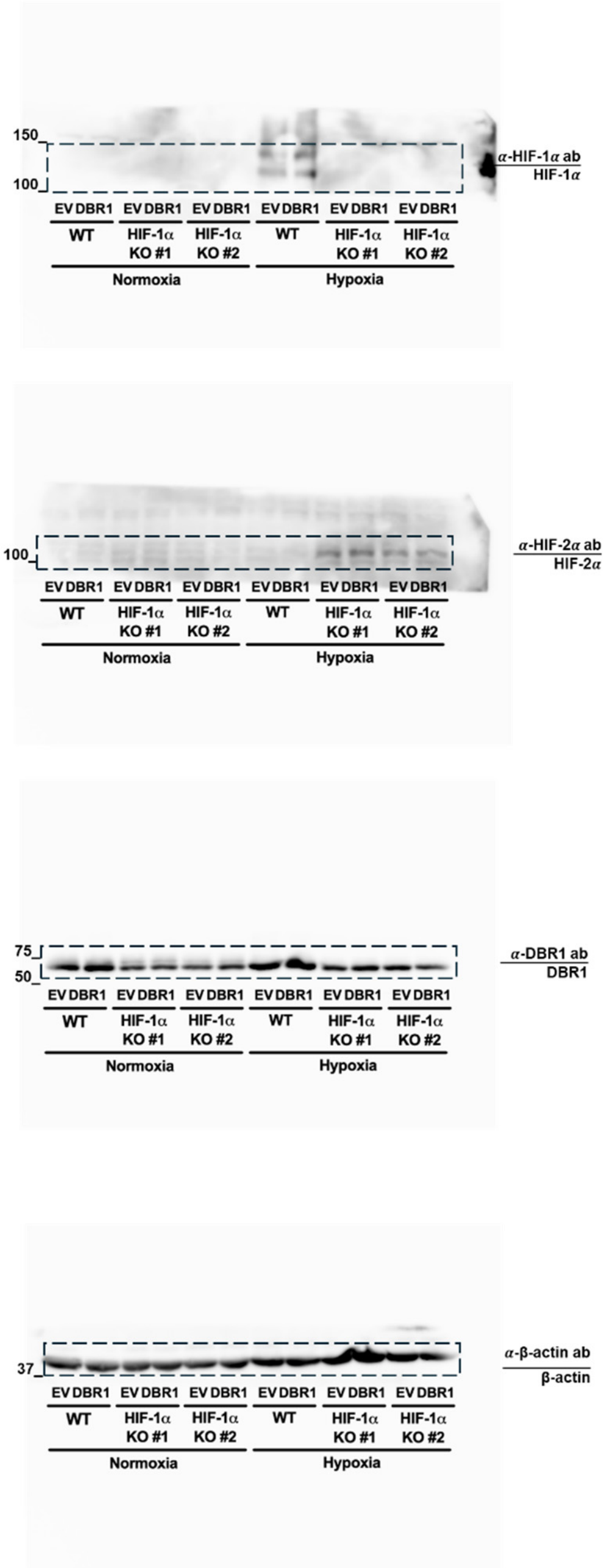

Sup. Fig. 6B

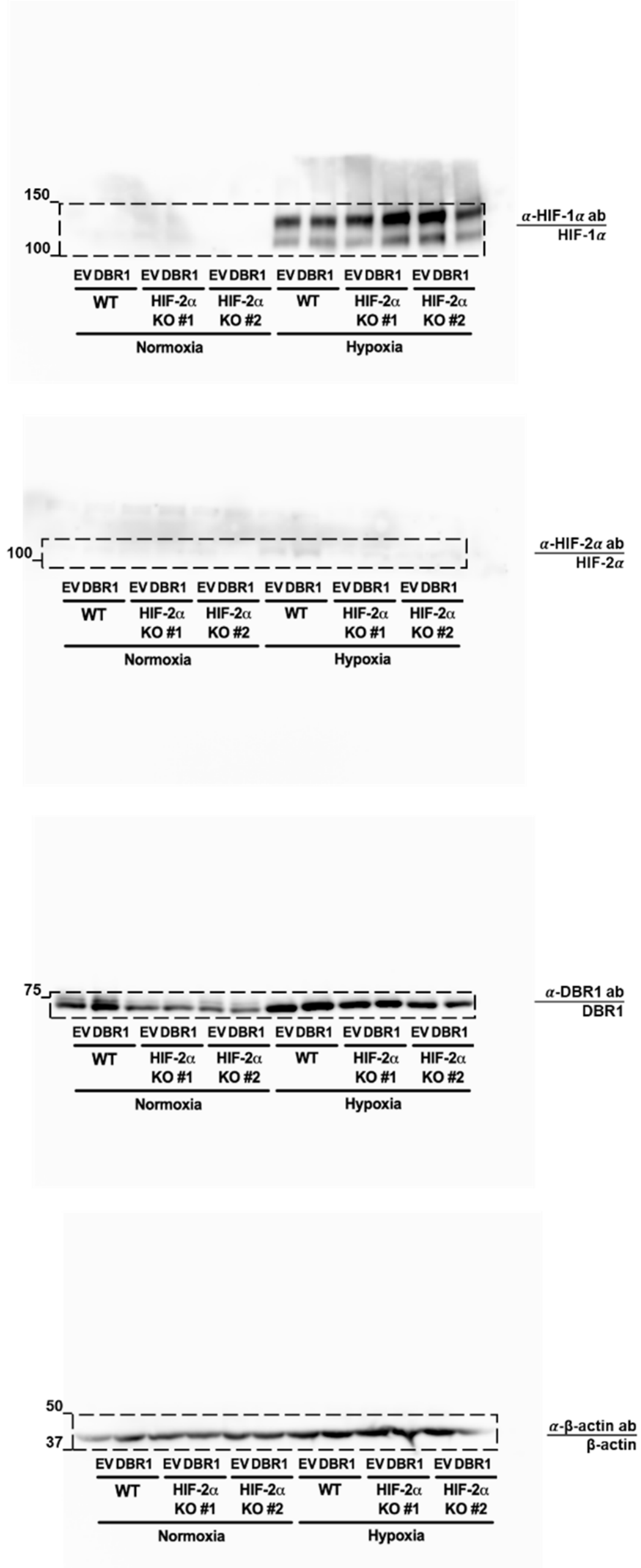

Supplementary Figure S11-8. Uncropped Western Blot Images

Sup. Fig. 6C

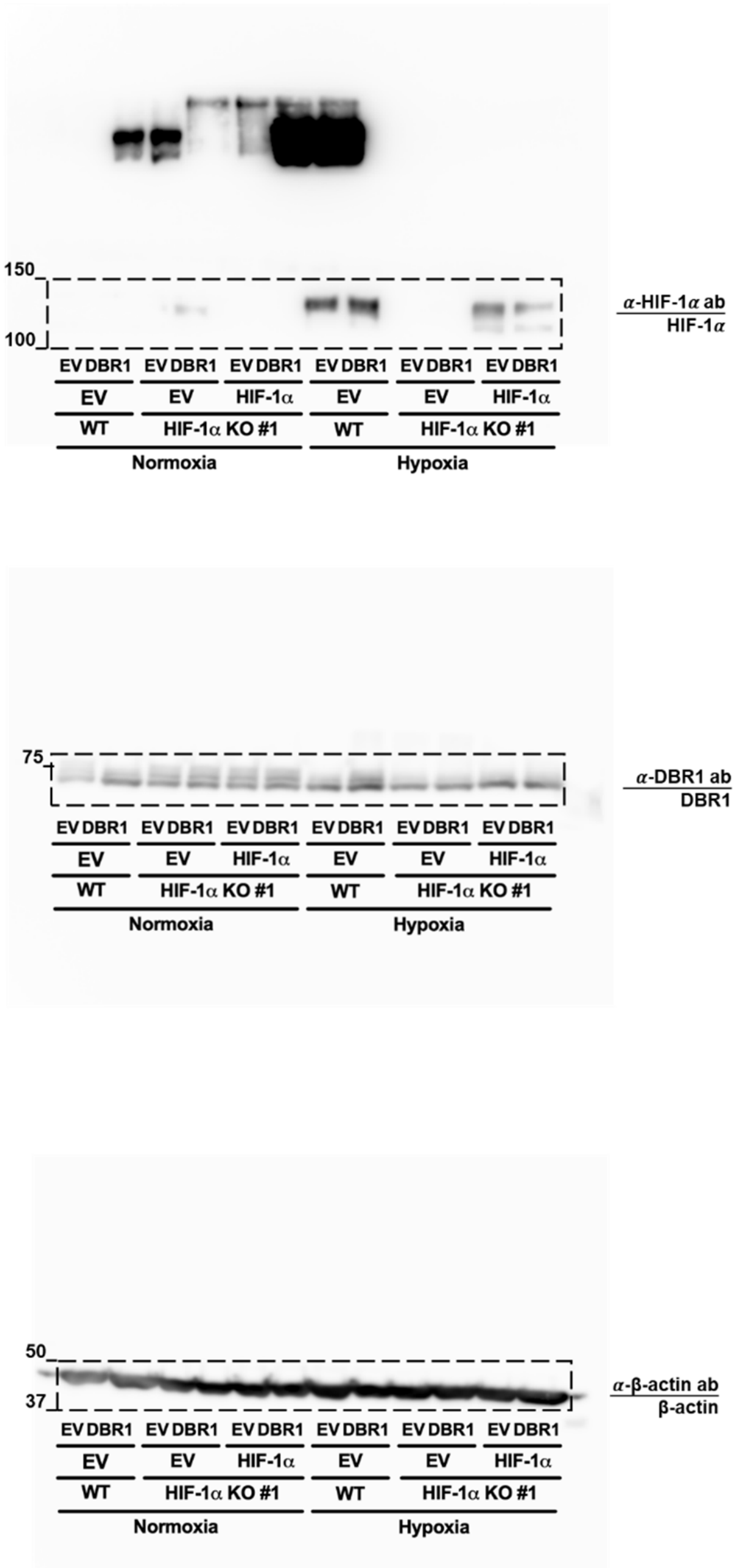

Sup. Fig. 9A

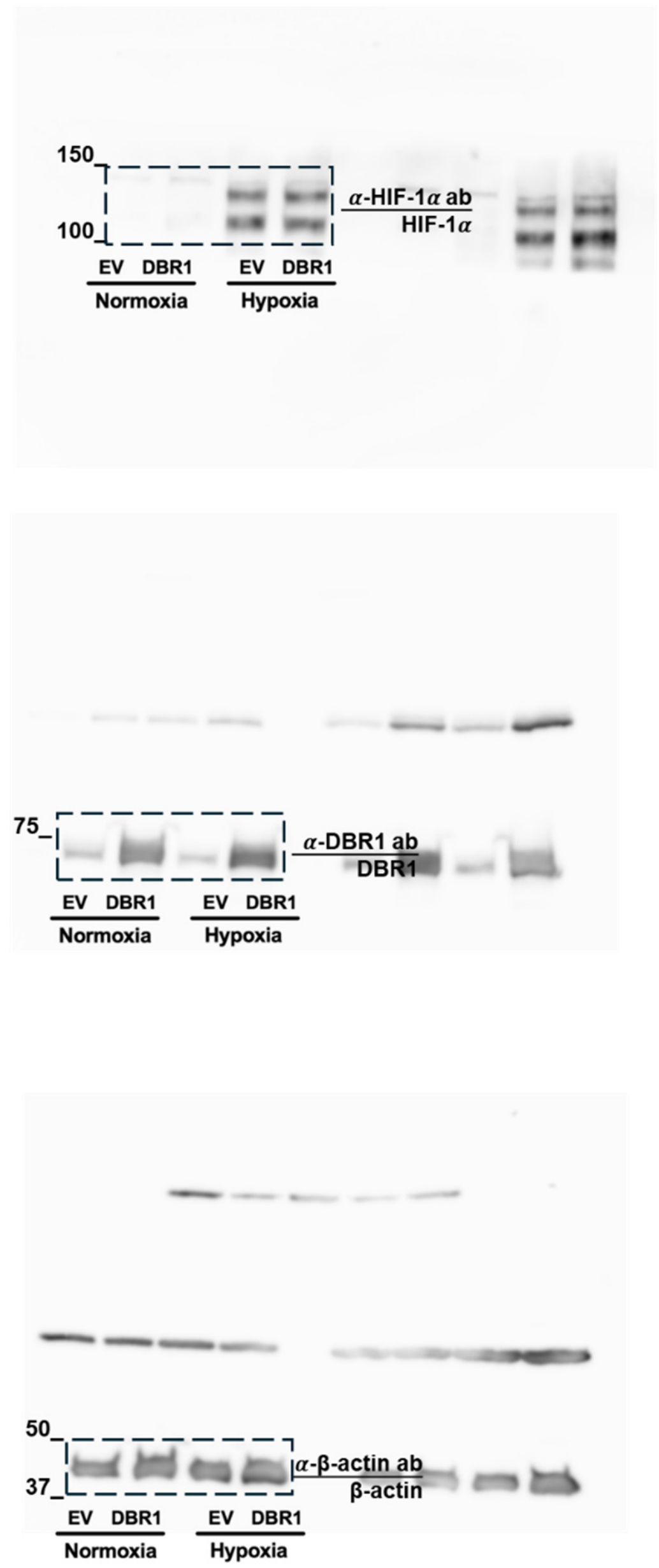

Supplementary Figure S11-9. Uncropped Western Blot Images

Sup. Fig. 9B

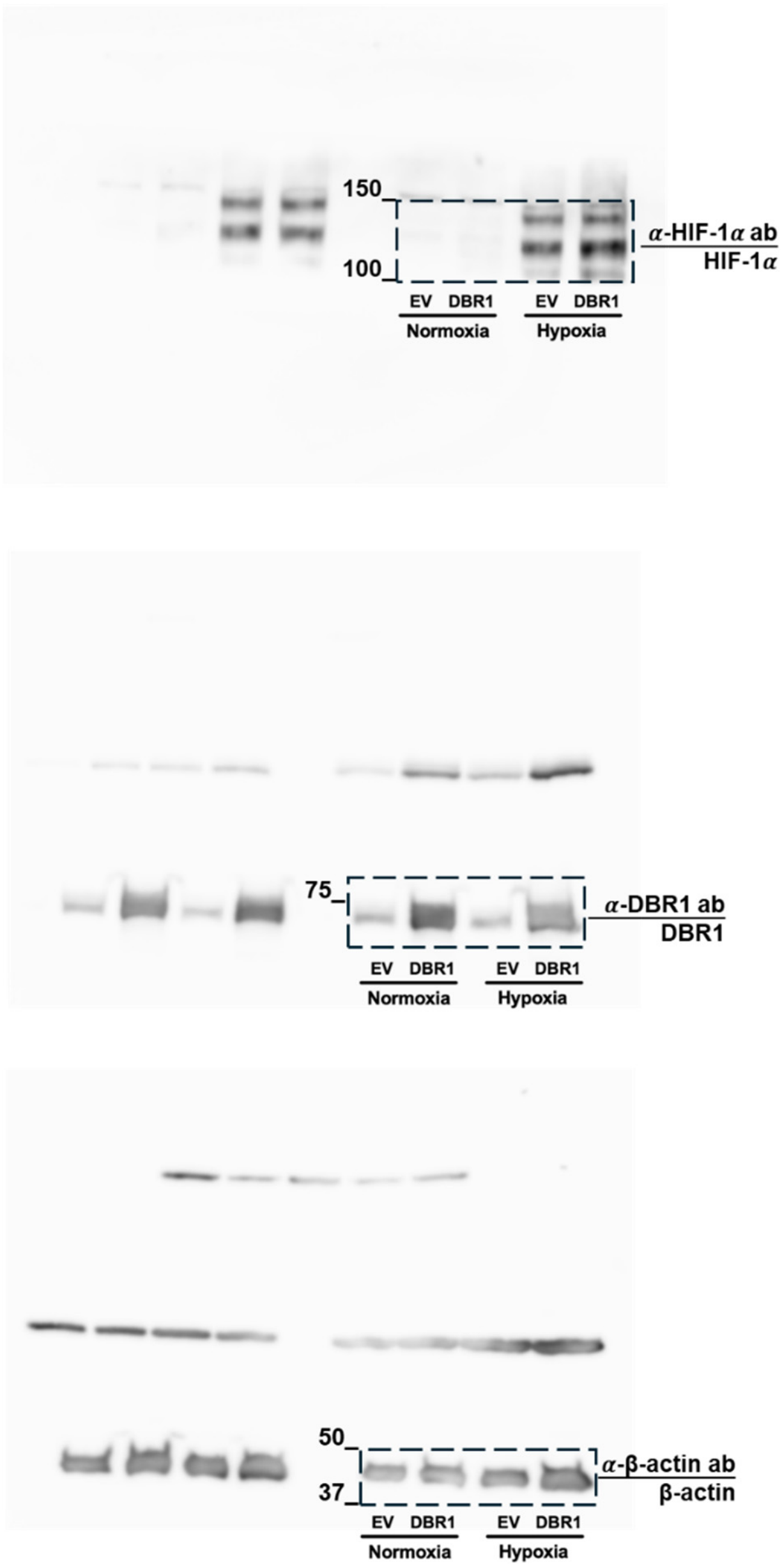

Sup. Fig. 9C

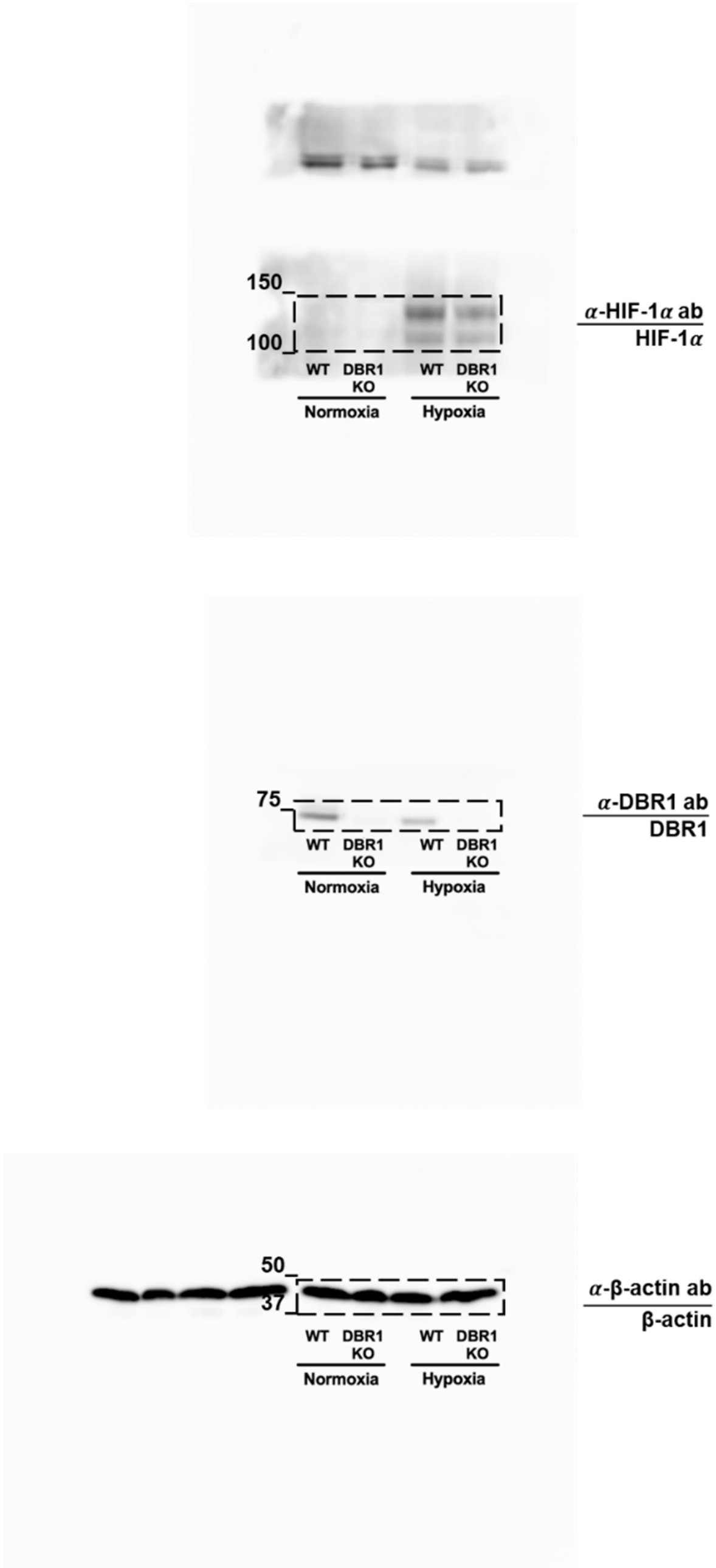

Supplementary Figure S11-10. **Uncropped Western Blot Images**

Sup. Fig. 9D

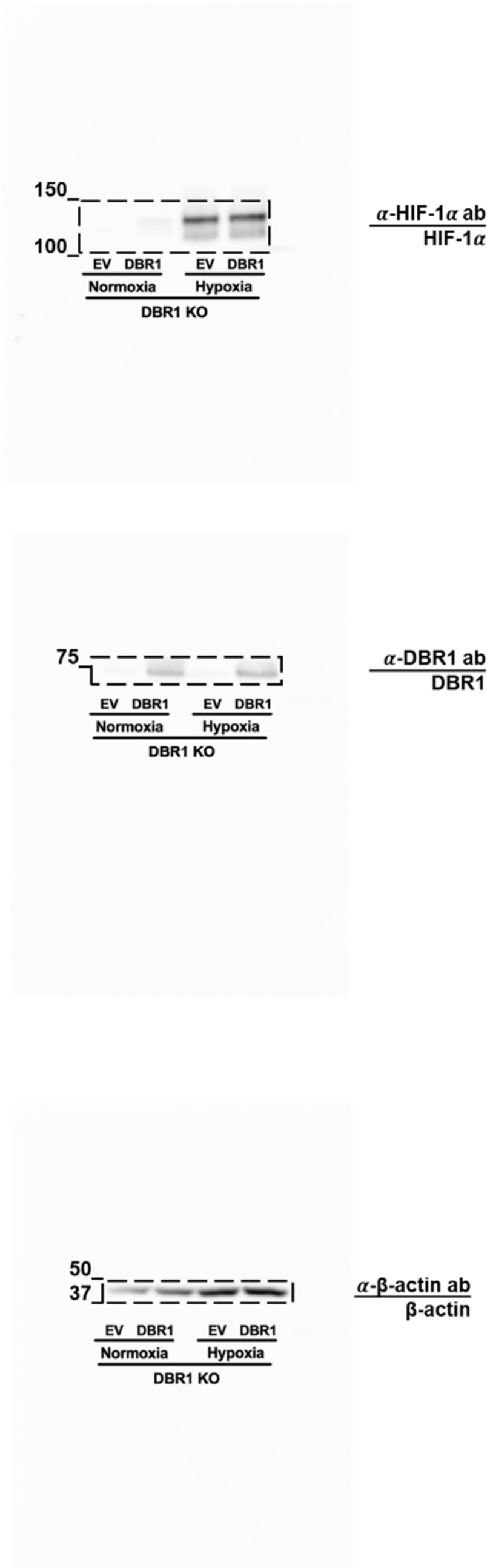

Sup. Fig. 10A

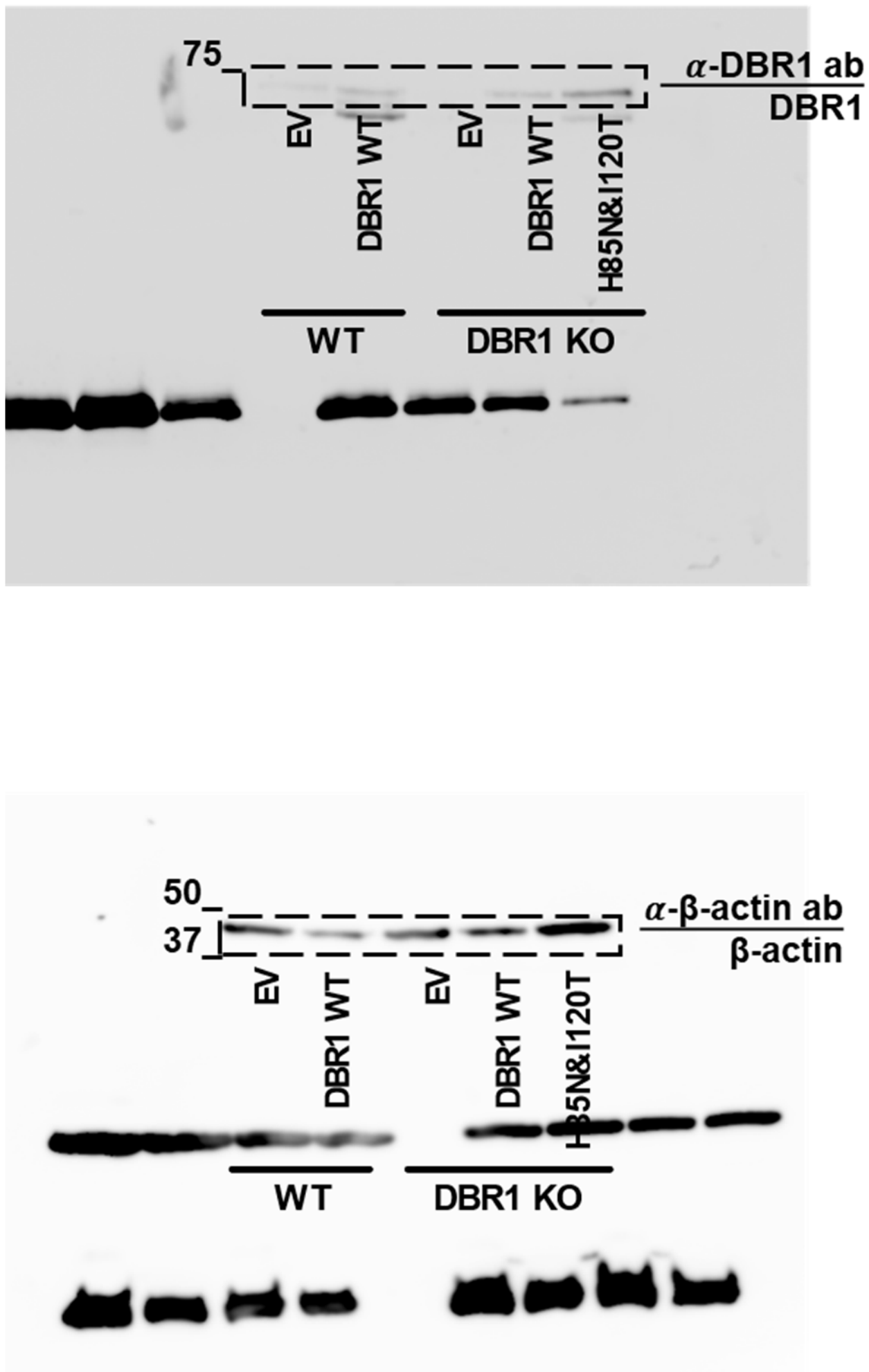

Supplementary Figure S11-11. **Uncropped Western Blot Images**

Sup. Fig. 10B

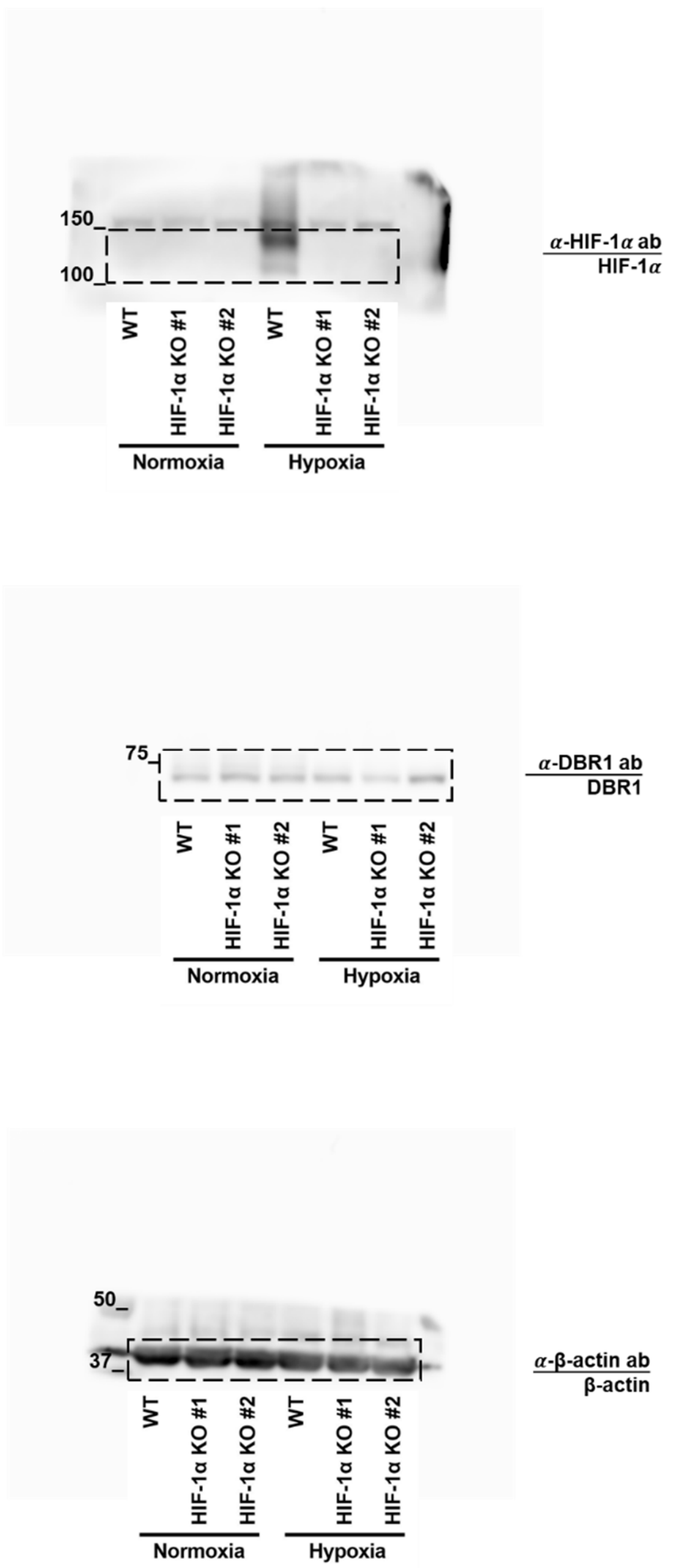

Sup. Fig. 10C

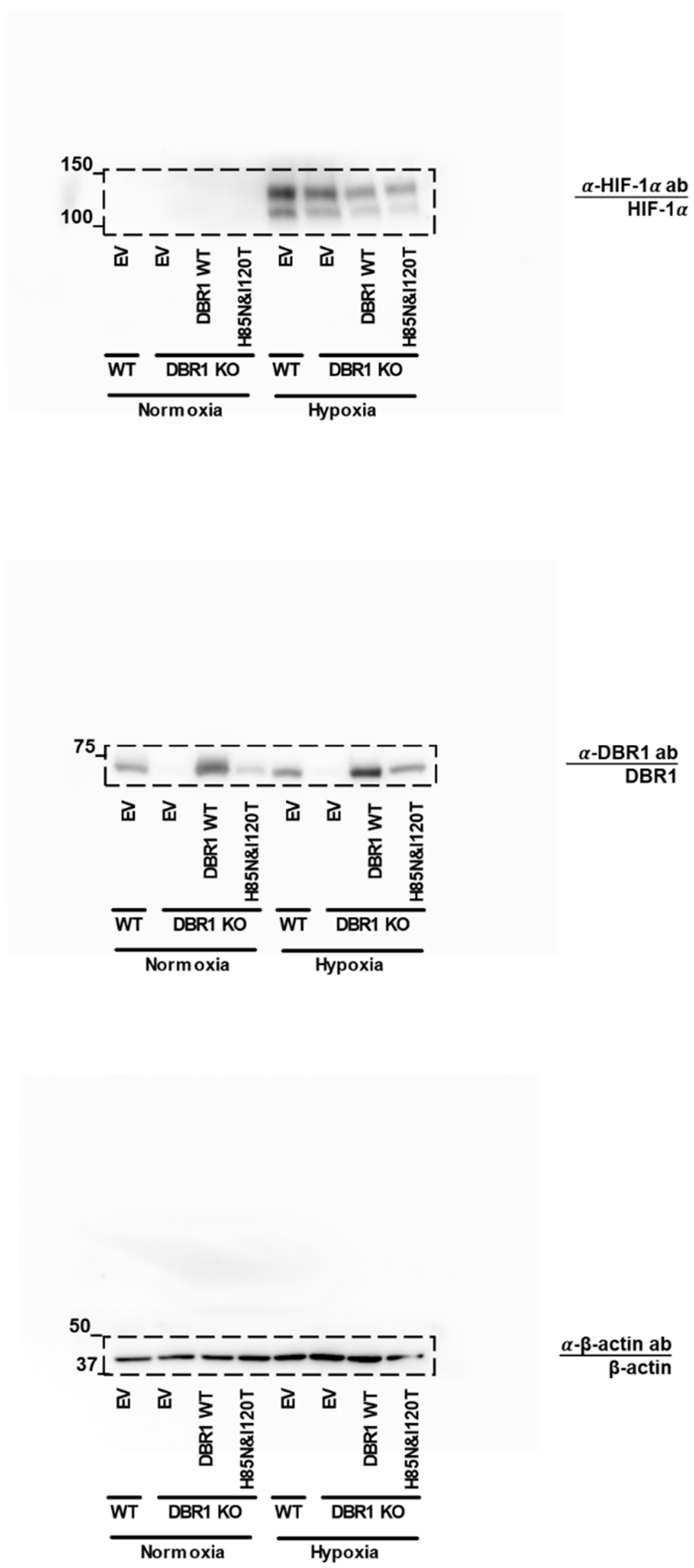

Supplementary Figure S11-12. **Uncropped Western Blot Images**
