# Supplementary material for: The lariat RNA debranching enzyme DBR1 enhances HIF-1 signaling and chemoresistance at the expense of lariat RNA processing
Source: J Biol Chem. 2026 Aug 25;302(10):113484. doi: 10.1016/j.jbc.2026.113484 (PMC13629192; doi:10.1016/j.jbc.2026.113484)
Supplement: Supplementary file 2 [file mmc2.pdf]

Colony No.	Multiplicity of infection	Target gene (s)
1	1	Mutated barcode
2	2	TAGLN, LY86
3	2	TAGLN, LY86
4	2	TAGLN, LY86
5	2	TAGLN, LY86
6	2	TAGLN, LY86
7	1	SEC31A
8	2	TAGLN, LY86
9	1	DBR1
10	2	MLH1, VCAN
11	2	OR51M1
12	2	TAGLN, LY86
13	2	TAGLN, LY86
14	2	TAGLN, LY86
15	2	TAGLN, LY86
16	2	TAGLN, LY86
17	1	OR51M1
18	2	TAGLN, LY86

Table S1. Candidate HIF regulators identified by shRNA screening.

Eighteen single-cell clones exhibiting reduced EGFP fluorescence under hypoxic conditions were isolated from the primary shRNA screen. Integrated shRNA sequences were identified by Sanger sequencing, and the corresponding target genes are listed. Candidate genes were sequentially subjected to secondary validation by luciferase reporter assays. DBR1, MLH1, VCAN, TAGLN, and SEC31A were evaluated in the secondary screen. After DBR1 was identified as a promising candidate for further investigation, the secondary screen was discontinued; therefore, OR51M1 and LY86 were not subjected to secondary validation.

Gene	Target sequences 5'-3'	
	shRNA #1	shRNA #2
DBR1	CCTGTCTTGGCCTTCTAAAGT	GCAAACCTTCTACAGGTATTA
MLH1	GCAGACTATTTCTCTTTGGAA	GTGTTCTTCTTTCTCTGTATT
VCAN	CCTGTGCCTTTCCTATCACCT	CCGATATTGATAGAGAGTATT
TAGLN	GCAGGAGCATAAGAGGGAATT	CCAACTTCTTACCCGAAAGCA
SEC31A	CTCAGAACCCTGGTGCTTTAT	GCTGATATACTCACCTTAGAA

Table S2. shRNA target sequences used for secondary validation of candidate HIF regulators.

The sequences of shRNAs used to individually evaluate the effects of the indicated candidate genes on HIF-dependent transcription are listed. Two independent shRNAs were used for each candidate gene.

Target		Sequence 5'-3'
β-actin mRNA	F	TGGCACCCAGCACAATGAA
	R	CTAAGTCATAGTCCGCCTAGAAGC
RPS18 mRNA	F	GCAGAATCCACGCCAGTACAAG
	R	GCTTGTTGTCCAGACCATTGGC
MET mRNA	F	TGAAATTCATCCAACCAAATCTT
	R	AATAGAAAACTGACAATGTTGAGAGG
HIF-1α mRNA	F	TCATCCAAGAAGCCCTAACGTG
	R	TTTCGCTTTCTCTGAGCATTCTG
5HRE in p5HRE-luc	F	TCTGGTAGGCGTGTACGGTG
	R	GCGCCGGGCCTTTCTTTATG
CA9 genome HRE	F	TCTCGTTTCCAATGCACGTACAGC
	R	AGTGACAGCAGCAGTTGCACAGT
ID1 mRNA	F	AAACGTGCTGCTCTACGACA
	R	CTCCAACTGAAGGTCCCTTGA
ID1 intron lariat	F	CCACTTCCGTCCCATCCTT
	R	GGTCGGATCTGGATCTCACTTGG
DKK1 mRNA	F	GAGCTACCCGGGTCTTTGTC
	R	GGGCAGGTTCTTGATAGCGT
DKK1 intron 1 lariat	F	GAGGGAGTAGAACGTGCTGA
	R	GCCCGACCCCTCTCACTGAG
ANGPTL4	F	CAGGCTACCTAAGAGGATGAGCGGTG
	R	CTGGCCGAGCTGCAGGAGTCCGTGC
CA9	F	ACCAGACAGTGATGCTGAGTGCTAA
	R	TCAGCTGTAGCCGAGAGTCACC
VEGFA	F	TTGCCTTGCTGCTCTACCTCCA
	R	GATGGCAGTAGCTGCGCTGATA
GLUT1	F	CTGCTCATCAACCGCAAC
	R	CTTCTTCTCCCGCATCATCT
BNIP3	F	GCTCCCAGACACCACAAGAT
	R	GCTACAATAGGCATCAGTCTGACA

Table S3. Primer sequences used for qRT-PCR and ChIP-qPCR analyses.

Forward (F) and reverse (R) primer sequences used for qRT-PCR and ChIP-qPCR analyses are listed.
